# Assessment of research waste part 2: wrong study populations- an exemplar of baseline vitamin D status of participants in trials of vitamin D supplementation

**DOI:** 10.1186/s12874-018-0555-1

**Published:** 2018-10-03

**Authors:** Mark J. Bolland, Andrew Grey, Alison Avenell

**Affiliations:** 1 Department of Medicine, Bone and Joint Research Group, University of Auckland, Private Bag 92 019, Auckland, 1142 New Zealand; 2 Health Services Research Unit, University of Aberdeen, Foresterhill, Aberdeen, AB25 2ZD Scotland

**Keywords:** Vitamin D, Deficiency, Sufficiency, Randomized controlled trials, Research waste, Fracture, Cardiovascular disease, Cancer, Mortality

## Abstract

**Background:**

Research waste can occur when trials are conducted in the wrong populations. Vitamin D deficient populations are most likely to benefit from vitamin D supplementation. We investigated waste attributable to randomised controlled trials (RCTs) of supplementation in populations that were not vitamin D deficient.

**Methods:**

In December 2015, we searched Pubmed, recent systematic reviews, and three trial registries for RCTs of vitamin D with clinical endpoints in adults, and 25-hydroxvitamin D (25OHD) survey data relevant to large (*N* ≥ 1000) RCTs. We investigated the proportion of RCTs that studied vitamin D deficient populations, temporal trends in baseline 25OHD, and whether investigators in large RCTs considered relevant 25OHD survey data or systematic reviews in their trial justifications.

**Results:**

Of 137 RCTs of vitamin D with clinical endpoints, 118 (86%) reported baseline mean/median 25OHD, which was < 25, 25–49, 50–74, and ≥ 75 nmol/L in 12 (10%), 62 (53%), 36 (31%), and 8 (7%) RCTs, respectively. In 70% of RCTs, baseline 25OHD was > 40 nmol/L. Baseline 25OHD increased over time. Before 2006, 38%, 62%, 0% and 0% of RCTs had baseline 25OHD < 25, 25–49, 50–74, and ≥ 75 nmol/L respectively; in 2011–15, the respective proportions were 9%, 49%, 37%, and 6%. Of 12 RCTs with baseline 25OHD < 25 nmol/L, 8 had neutral findings. Of 25 large RCTs (18 completed, 7 ongoing), 1 was undertaken in a vitamin D deficient population, 3 in vitamin D insufficient populations, and 17 had, or probably will have, baseline 25OHD > 40 nmol/L. 44% (8/18) of large completed RCTs cited relevant prior population 25OHD data, and only 3/10 (30%) relevant prior systematic reviews.

**Conclusions:**

Up to 70% of RCTs of vitamin D with clinical endpoints, 71% of large completed RCTs, and 100% of ongoing large RCTs could be considered research waste because they studied cohorts that were not vitamin D deficient.

**Electronic supplementary material:**

The online version of this article (10.1186/s12874-018-0555-1) contains supplementary material, which is available to authorized users.

## Background

Chalmers and Glasziou estimated that 85% of clinical research is wasteful, with 50% of studies having design or major methodological weaknesses [1]. In these companion reports, we assessed research waste in a single field - calcium and vitamin D research. In the first report [2], we focused on redundant research characterized by the undertaking and publication of uninformative observational studies and randomised controlled trials (RCTs) with surrogate endpoints long after the need for large RCTs with ‘hard’ clinical endpoints was established. In this second report, we address waste characterised by conducting RCTs in poorly targeted population groups.

Clinical guidelines disagree on the serum 25-hydroxyvitamin D (25OHD) concentrations that constitute vitamin D sufficiency. The Institute of Medicine recommends ≥50 nmol/L to ensure adequate 25OHD for 97.5% of the population, with a median target value of 40 nmol/L [3], whereas the Endocrine Society recommends ≥75 nmol/L [4]. However despite this disagreement, there is general agreement that 25OHD < 25 nmol/L indicates deficiency, and recent UK guidance on vitamin D supplementation is based on maintaining 25OHD above this threshold [5]. Mildly low 25OHD is often termed vitamin D insufficiency, and moderately low 25OHD vitamin D deficiency. Throughout the text, we have used vitamin D deficiency to refer to 25OHD < 25 nmol/L, and insufficiency to 25OHD < 50 nmol/L [6]. Intuitively, supplementing populations that are vitamin D deficient is more likely to produce benefits than supplementing populations with higher 25OHD [7]. Potential benefits of vitamin D could be obscured if a high proportion of participants in RCTs are vitamin D sufficient. Thus, RCTs in cohorts that are vitamin D sufficient are unlikely to show benefits of vitamin D supplementation and could be considered research waste. Recent systematic reviews of RCTs of vitamin D supplementation have not shown benefits on skeletal or non-skeletal endpoints [8–11]. We set out to determine what proportion of RCTs of vitamin D supplementation with clinical endpoints has been conducted in vitamin D deficient populations, and whether baseline 25OHD in such RCTs have changed over time. We then focused on large RCTs either already completed or in progress, identified data on target population vitamin D status available prior to the trial, and determined whether the investigators reported relevant data on vitamin D status. We also determined whether investigators reported relevant systematic reviews in their trial justification, as recommended [1, 12].

## Methods

### Literature searches

In December 2015, we searched Pubmed for RCTs of vitamin D in adults (>18y) (Additional file 1: Table S1) and for recent systematic reviews on clinical conditions and major surrogate endpoints that were the primary endpoints in identified RCTs (Additional file 1: Tables S2 and S3). We included trials with an untreated or placebo group, trials comparing different vitamin D doses, trials with or without calcium supplements, and trials with multiple interventions provided that 2 study arms differed only by the use of vitamin D. We included quasi-randomized trials but excluded trials where the method of allocation was sequential or unreported, trials where vitamin D was administered routinely post-thyroidectomy, and trials of hydroxylated vitamin D analogues. The flow of articles is shown in Additional file 1: Figure S1.

In December 2015, we also searched ClinicalTrials.gov (https://clinicaltrials.gov/), the International Standard Randomised Controlled Trial Number (ISRCTN) registry (http://www.isrctn.com/) and the Australian New Zealand Clinical Trials Registry (ANZCTR) (http://www.anzctr.org.au/) for completed and ongoing trials, using vitamin D as the search term.

Finally, we obtained vitamin D status survey data from published systematic reviews [13–17]. supplemented by Medline, Embase, and Google searches using our vitamin D search strategy and text words for the countries of interest, and checked citations in reference lists.

### Trial classification

We categorised each RCT according to whether clinical or surrogate endpoints were reported in the Abstract (or full-text where there was no Abstract), using the Institute of Medicine definition of surrogate outcomes as “biomarker[s] intended to substitute for a clinical endpoint [and] expected to predict clinical benefit (or harm. ..) based on epidemiologic, therapeutic, pathophysiologic, or other scientific evidence” [18]. Where multiple endpoints were reported, we recorded the most relevant clinical endpoint, and if there were no clinical endpoints, the most clinically relevant surrogate endpoint. Where there were multiple publications from the same RCT, we included the study with the most relevant clinical endpoint or the most clinically relevant surrogate endpoint.

### Vitamin D status survey data

For large (*N* ≥ 1000) completed and ongoing RCTs, we identified surveys of vitamin D status undertaken in the same country and most similar population group, based on age and sex, prior to the trial being undertaken. We preferentially sought data from the five years before trial inception or 10 years before trial completion/publication, but used older data if we could not locate such data.

### Analyses

A single author (MB or AA) extracted relevant data. One author (MB) classified trials as having clinical or surrogate endpoints, and a second author (AG) checked the classifications. We report the proportions of trials with mean/median baseline 25OHD < 25, 25–49, 50–74, ≥75 nmol/L over time. In trials with mean/median baseline 25OHD < 25 nmol/L and trials that reported a subgroup analysis based on baseline 25OHD, two authors (MB, AG) independently assessed whether the results of the trial or subgroup analysis were beneficial, neutral, or harmful and disagreements were resolved by consensus.

We examined primary trial publications, and trial protocols (where available), for large RCTs (*N* ≥ 1000) and assessed whether trial investigators discussed prior relevant evidence on vitamin D status of the intended trial population in their justification for the trial. We also examined whether trial investigators discussed systematic reviews of randomised trials relevant to the primary endpoint that were available before trial recruitment commenced in the Introduction section of the primary publication.

Early 25OHD competitive binding protein (CBP) assays overestimated 25OHD concentrations [19]. As an approximation, we used an adjustment factor of 0.54 for CBP assays in papers published before 2000 [19]. and 0.76 to adjust for overestimation from the Nicholls’ immunoassay [20]. We have presented the RCT and survey data in Tables 3 and 4 corrected for these overestimations.

## Results

### Baseline 25OHD in randomised controlled trials

**Fig. 1:**
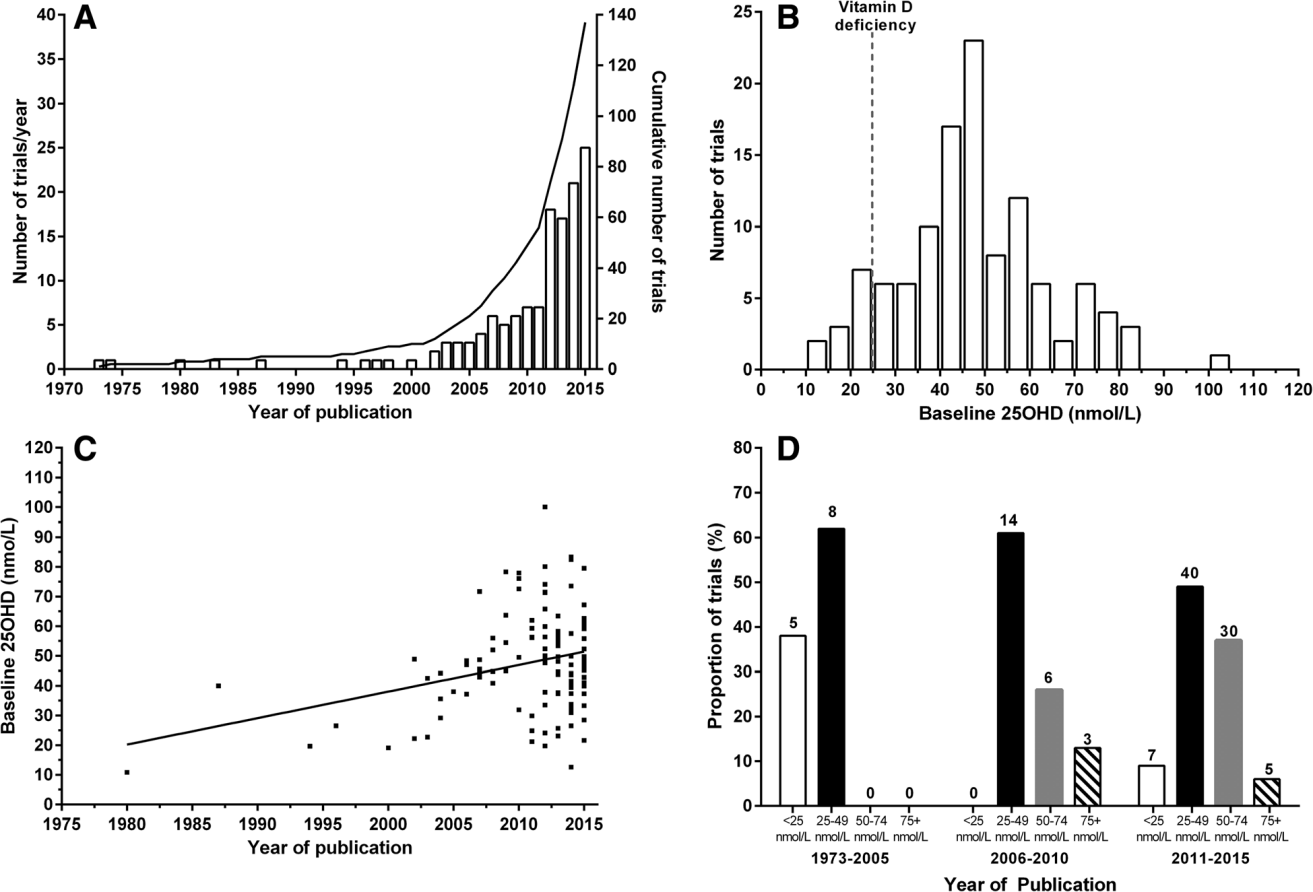
Panel **a** shows the number of randomized controlled trials (RCTs) of vitamin D with clinical endpoints in the Abstract published over time by year (bars) and cumulatively (line). Panel **b** shows the distribution of mean/median baseline 25-hydroxyvitamin D (25OHD) concentrations in these RCTs. Panel **c** shows the 25OHD concentrations plotted against year of publication with a line of best fit. Panel **d** shows the proportion of trials with mean/median baseline 25OHD < 25, 25–49, 50–74 and ≥ 75 nmol/L by year of publication. Above each bar is the number of trials

From 4682 unique Pubmed records and 38 systematic reviews, we identified 779 publications from 547 RCTs of vitamin D, of which 137 (111,976 participants) reported a clinical endpoint in the Abstract (Additional file 1: Tables S1, S2, S3 and Figure S1). Figure 1a shows that the rate of publication of RCTs has increased markedly, with 11 RCTs in 2001–5, 28 in 2006–10, and 88 in 2011–15. Mean/median baseline 25OHD was reported in 118/137 (86%) RCTs (Fig. 1b), with 62%, 82%, and 93% of RCTs reporting baseline 25OHD before 2006, in 2006–10, and in 2011–15 respectively. Overall, mean/median baseline 25OHD was < 25, 25–49, 50–74, and ≥ 75 nmol/L in 12 (10%), 62 (53%), 36 (31%), and 8 (7%) RCTs, respectively. In 70% of RCTs, baseline 25OHD was > 40 nmol/L. Of 12 RCTs with baseline 25OHD < 25 nmol/L, 8 had neutral findings (Table 1).

**Table 1:** Characteristics of 12 randomised controlled trials of vitamin D supplements in populations with mean/median 25OHD < 25 nmol/L and clinical endpoints reported in abstract

Study	Clinical endpoint	Endpoint type	Study Size (N)	25OHD Assay	Mean/Median 25OHD (SD) (nmol/L)^a^	Result of Trial^b^
Brooke 1980 [44]	Newborn outcomes	Secondary	126	CBP	11 (1)	Benefit
Chapuy 1994 [21]	Fracture	Primary	3270	CBP	20 (14)	Benefit
Pfeifer 2000 [45]	Risk of fall	Secondary	148	Nicholls	19 (10)	Neutral
Chapuy 2002 [46]	Fracture	Secondary	583	Incstar	22 (16)	Neutral
Bischoff 2003 [47]	Risk of fall	Primary	122	Nicholls	23 (N/A)	Neutral
Martineau 2011 [48]	Tuberculosis sputum culture conversion	Primary	126	LCMS/MS	21 (20)	Neutral
Mosayebi 2011 [49]	Multiple sclerosis disability score	Primary	59	IDS	25 (7)	Neutral
Amestejani 2012 [50]	Atopic dermatitis	Primary	60	Biosource	24 (5)	Benefit
Schreuder 2012 [51]	Pain	Primary	84	Diasorin	20 (10)	Neutral
Mozaffari-Khosravi 2013 [52]	Depression score	Primary	120	IDS	23 (N/A)	Benefit
Hossain 2014 [53]	Pregnancy outcomes	Primary	200	Immunoassay	13 (N/A)	Neutral
Bhan 2015 [54]	All-cause mortality	Secondary	105	LCMS/MS	22 (7)	Neutral

^a^Adjusted for assay- see text for details

^b^Based on intention-to-treat analysis of all randomized participants for relevant endpoint. Assessed independently by two authors (MB, AG)

Studies are listed in Additional file 1: Table S3 and the Additional file 1: Reference list

*Abbreviations*: *25OHD* 25-hydroxyvitamin D, *SD* standard deviation, *N/A* not available. *CBP* competitive binding protein; LCMS/MS- liquid chromatography tandem mass-spectrometry

Figure 1c and d show that mean/median baseline 25OHD has increased over time. Before 2006, 38% of RCTs had 25OHD < 25 nmol/L, 62% between 25 and 49 nmol/L, and none ≥50 nmol/L. In 2006–10 and 2011–15, 0% and 9% respectively of RCTs had 25OHD < 25 nmol/L, while 61% and 49% respectively had 25OHD 25–49 nmol/L, 26% and 37% respectively had 25OHD 50–74 nmol/L, and 13% and 6% respectively had 25OHD ≥75 nmol/L.

Of 118 RCTs that reported mean/median baseline 25OHD, 19 (16%) reported a subgroup analysis for baseline 25OHD (Table 2). The 25OHD thresholds used in these analyses ranged from 20 to 80 nmol/L, with 5 analyses based on thresholds ≤25 nmol/L and 16 on thresholds ≤50 nmol/L. Table 2 shows that 17 RCTs reported similar results in the subgroup analysis and the main analysis for the primary endpoint (16 both analyses neutral, and 1 both analyses showed benefit for vitamin D). Three of these 17 RCTs reported a benefit for vitamin D for a secondary endpoint in a subgroup analysis. Another RCT did not report the result of the subgroup analysis for the primary endpoint, but reported a benefit for vitamin D for a secondary endpoint. Lastly, one RCT had co-primary endpoints and neutral results in the main analyses, but in the subgroup analyses there was a benefit for vitamin D for one endpoint and neutral results for the other. Four of the five RCTs that reported subgroup analyses with a 25OHD threshold of ≤25 nmol/L had neutral results for the primary endpoint in the main analysis, and none of these 4 RCTs reported beneficial effects for the primary endpoint in the subgroup analysis.

**Table 2:** Results of 18 randomised controlled trials of vitamin D supplements reporting subgroup analyses for baseline 25-hydroxyvitamin D

Study	25OHD threshold (nmol/L)	Subgroup Result^a^	Comparison to primary analysis^a^
Jackson 2006 [33]	32.2	Neutral	Same
Jorde 2008 [55]	40	NR	N/A^b^
Wejse 2009 [56]	75	Neutral	Same
Martineau 2011 [48]	20	Neutral	Same
Rastelli 2011 [57]	50	Neutral	Same^c^
Kjaergaard 2012 [58]	25	Neutral	Same
Lehouck 2012 [59]	25	Neutral	Same^b^
Murdoch 2012 [60]	50	Neutral	Same
Abou-Raya 2013 [61]	25	Benefit	Same
McAlindon 2013 [62]	37.5	Neutral	Same
Amrein 2014 [63]	30	Neutral	Same^b^
Lopez-Torres Hidalgo 2014 [64]	80	Neutral	Same
Tran 2014 [65]	50	Neutral	Same
Turner 2014 [66]	50	Neutral	Same
Baron 2015 [36]	57.9	Neutral	Same
Martineau 2015 [67]	50	Benefit	Different^d^
Miskulin 2015 [68]	37.5	Neutral	Same
Sandoughi 2015 [69]	50	Neutral	Same
Tukvadze 2015 [70]	25	Neutral	Same

^a^ Assessed independently by two authors (MB, AG)

^b^ Benefit for secondary endpoint in subgroup analysis

^c^ Primary endpoint not specified. Benefits in subgroup analyses for some but not all reported endpoints

^d^ Two co-primary endpoints. Benefit in subgroup analysis for one co-primary endpoints. For other co-primary endpoint, subgroup analysis was neutral. In primary analyses, results for both co-primary endpoints were neutral

Studies are listed in Additional file 1: Table S3 and Reference list

*Abbreviations: 25OHD* 25-hydroxyvitamin D, *NR* not reported; N/A not applicable

### Large randomised controlled trials and prior 25OHD surveys

Tables 3 and 4 show 18 published RCTs of vitamin D with ≥1000 participants (101,383 participants), and 7 planned and ongoing trials (79,939 intended participants). We included the pilot stage for the UK VIDAL trial with 1600 participants, which aimed to continue and recruit 20,000 participants, but has not yet received funding for the full roll out. All trials were/are conducted exclusively in North America, Europe, Australia or New Zealand, except for two multinational trials with countries from South America, Asia and Africa. 22/25 trials were in single countries: we did not examine prior 25OHD surveys for the 3 multinational trials.

**Table 3:** Large randomised controlled trials of vitamin D supplements with relevant prior 25-hydroxyvitamin D surveys

Trial					Survey identified			
Reference/ Country	Trial characteristixcs	Baseline 25OHD (nmol/L) /Assay^a^	Cites Prior 25OHD Survey (S)/ Prior SR (PSR) /Any SR (ASR)	Recruit-ment started	Survey Date	Group surveyed	25OHD [mean (SD)] (nmol/L)/Assa^a^	Survey Reference^b^
Chapuy 1994 [21] France	*N* = 3270 100% Female Mean age 84y	Subg 20 CPB	S:Yes Chapuy 1987 PSR:No^e^ ASR:No	NS	1984	Men and women Mean age 74-75y	CPB	Chapuy 1987 [71]
						Outpatients	23 (10)	
						Long stay hospital	11 (6)	
Lips 1996 [22] Netherlands	*N* = 2578 74% Female Mean age 80y	Subg 27 HPLC	S:Yes Lips 1987 PSR:No^e^ ASR:No	1988	NS	Men and women Mean age 76y	CPB	Lips 1987 [72]
						Hip fracture patients	10.0 (5.7)	
						Apartment dwellers	17.8 (7.3)	
					1984–5		CPB	Lowik 1990 [73]
						Men 65-79y	21.6 (10.3)	
						Women 65-79y	20.5 (8.6)	
					NS	Men and women Mean age 81-84y	CPB	Lips 1988 [74]
						Nursing home	12.7 (4.8)	
						Aged people home	12.9 (7.2)	
Meyer 2002 [23] Norway	*N* = 1144 76% Female Mean age 85y	Subg 49 HPLC	S:Yes Mowe 1998 PSR:No^e^ ASR:Yes	1995	1989	Men and women Mean age 78-80y Hospital patients	HPLC	Mowe 1998 [75]
						Men	40.4 (23.2)	
						Women	37.5 (22.6)	
						Home-living		
						Men	59.6 (28.9)	
						Women	48.5 (20.3)	
					1989		CPB	Nes 1993 [76]
						Men 75-76y	24.1 (10.1)	
						Women 75-76y	25.9 (11.2)	
Trivedi 2003 [37] UK	*N* = 2686 24% Female Mean age 75y	ND	S:No PSR:No^e^ ASR:No	1996	1994-5	Men and women	Incstar	Finch 1998 [77]
						Free-living 65y+	55.5 (26.9)	
						Institution 65+	32.8 (15.7)	
Larsen 2004 [24] Denmark	*N* = 9605 60% Female Mean age 75y	Subg 36 Diasorin	S:Yes Lund 1979 PSR:No^e^ ASR:Yes	1995	Pre 1979	Men and women	CPB	Lund 1979 [78]
						61-93y	26.8 (12.4)	
					1989	Men and women	CPB	van der Wielen 1995 [14]
						75-81y	Men 24 Women 22	
Grant 2005 [29] UK	*N* = 5292 85% Female Mean age 77y	Subg 38 HPLC	S:No PSR:Yes ASR:Yes	1999	1994–5	Men and women	Incstar	Finch 1998 [77]
						Free-living 65y+	55.5 (26.9)	
						Institution 65+	32.8 (15.7)	
Porthouse 2005 [38] UK	*N* = 3314 100% Female Mean age 77y	ND	S:No PSR:No ASR:No	2001	1994–5	Women	Incstar	Finch 1998 [77]
						Free-living 65y+	51.7 (24.7)	
						Institution 65+	32.5 (15.5)	
Jackson 2006 [33] USA	*N* = 36,282 100% Female Mean age 62y	Subg 48 Liaison	S:No PSR:No^e^ ASR:Yes	1995	1988-94	Men and women	LC-MS/MS equivalent	Schleicher 2016^c^ [79]
						40-59y	60.1 (58.7,61.5)	
						≥60y	58.4 (57.4,59.5)	
						All females	59.2 (57.9,60.6)	
Law 2006 [34] UK	*N* = 3717 76% Female Mean age 85y	Subg 47 IDS	S:No PSR:No ASR:No	2000	1994–5	Men and women	Incstar	Finch 1998 [77]
						Institution 65+	32.8 (15.7)	
Lyons 2007 [39] UK	*N* = 3440 76% Female Mean age 84y	ND	S:No PSR:No ASR:Yes	1999	1994–5	Men and women	Incstar	Finch 1998 [77]
						Institution 65+	32.8 (15.7)	
Smith 2007 [35] UK	*N* = 9440 54% Female Mean age 79y	Subg 43 Nicholls	S:No PSR:No ASR:Yes	1998	1994–5	Men and women	Incstar	Finch 1998 [77]
						Free-living 65y+	55.5 (26.9)	
						Institution 65+	32.8 (15.7)	
Lappe 2008 [25] USA	*N* = 5201 100% Female Median age 19y	ND	S:Yes Gordon 2004 PSR:No ASR:No	2001	2001–3	Boys and girls	Nichols	Gordon 2004 [80]
						11-18y		
						Summer	49.8 (21.3)	
						Winter	38.2 (18.8)	
					2001–2	Males and females	LC-MS/MS equivalent	Schleicher 2016 [79]
						12-19y	63.0 (60.8,65.2)	
						20-39y	62.8 (60.6,64.9)	
					1988–94	Males and females	LC-MS/MS equivalent	Schleicher 2016^c^ [79]
						12-19y	66.2 (64.1,68.4)	
						20-39y	64.4 (62.8,66.0)	
Salovaara 2010 [30] Finland	*N* = 3432 100% Female Mean age 67y	Subg 50 Diasorin	S:No PSR:No ASR:Yes	2002	2000–1	Women	Incstar	Kauppi 2009 [81]
						Mean age 53y Age range 30-97y	45.2 (26.4)	
Sanders 2010 [26] Australia	*N* = 2258 100% Female Mean age 76y	Subg 50 Diasorin	S:Yes Pasco 2001 PSR:No ASR:Yes	2003	1994–7	Women	Incstar	Pasco 2001 [82]
						60-79y	62 (31.7)	
						80y+	53 (26.8)	
Punthakee 2012 [42] Multinational	*N* = 1221 41% Female Mean age 67y	ND	S: No PSR: Yes ASR:Yes	2009				
Baron 2015 [36] USA	*N* = 2259 37% Female Mean age 58y	61 IDS	S:No PSR:No^e^ ASR:Yes	2004	2001-2	Men and women	LC-MS/MS equivalent	Schleicher 2016^c^ [79]
						40-59y	62.4 (59.9,64.8)	
						≥60y	60.4 (58.0,62.9)	
Cooper 2016 [27]^d^ UK	*N* = 1134 100% Female Mean age 31y	47 Liaison	S:Yes Javaid 2006 PSR:No^e^ ASR:Yes	2008	1991-2	Pregnant women	IDS	Javaid 2006 [83]
						Mean 27y	18% < 26.5 31% 26.5–50 52% > 50	
					2008–12	Women 19 - 64y	Liaison 47.3	National Diet and Nutrition Survey 2014. [84]
ViDA 2017 [28]^d^ New Zealand	*N* = 5110 42% Female Mean age 66y	63 LCMS/MS	S:Yes Rockwell 2006 PSR:Yes ASR:Yes	2011	1996–7	Men 45-64y/65y+	52/55	Rockell 2006 [85]
						Women 45-64y/65y+	45/43 Diasorin	
					2008–9	Men and women	61/63/66/62	Adult nutrition survey 2009 [86]
						45-54y/55-64y/65-74y/≥75y	LCMS/MS

**Table 4:** Planned and ongoing large randomised controlled trials of vitamin D supplements with relevant prior 25-hydroxyvitamin D surveys

Trial				Survey identified			
Trial/Country	Trial details	Cites 25OHD Survey (S)/ Systematic Review (SR)	Recruitment started	Survey Date	Group surveyed	25OHD[mean/median(SD)] (nmol/L)/Assay	Survey Reference^a^
D-Health Australia	*N* = 21,315, 5y 60,000 IU D3 monthly v placebo Men/women 60-84y ACTRN12613000743763	S:Yes Tran 2012 pilot PSR:Yes ASR:Yes	2014	2010–1	Men and women	41.7 (13.5)	Tran 2012 [87] Waterhouse 2015 [88]
					Mean age 72y	Liaison	
				2011–2	Men and Women	68.9/69.8/68.6	Australian health survey 2011–2 [89]
					55-64y/65-74y/>75y	LCMS/MS	
DO-HEALTH 5 countries in Europe	*N* = 2152, 3y 2000 IU/d D3 v placebo Men/women ≥70y NCT01745263	S:NDA PSR:NDA ASR: NDA	2012				
FIND Finland	*N* = 2495, 5y 1600 IU/d D3 v 3200 IU/d D3 v placebo Men ≥60y, women ≥65y NCT01463813	S:Yes Hurskainen 2012 PSR:No ASR:Yes	2012	1998- 2001	Men and women	43.4 (17.6)	Hurskainen 2012 [90]
					Mean age 62.9y	HPLC	
	Recruitment stopped early had aimed for 18,000			2003–5	Men and women	64.8 (17.4)	Salminen 2015 [91]
					Mean age 73.5y	IDS	
				2011–2	Men and women	58.6 (9.3)	Carlberg 2013 [92]
					Mean age 66.6y	HPLC	
TIPS-3 10 countries in Africa, Asia, South/ North America	*N* = 5000, 5y 60,000 IU D3 3 monthly v placebo Men ≥55y, women ≥60y NCT01646437	S:NDA PSR:NDA ASR:NDA	2012				
VIDAL UK	*N* = 1600, 2y 100,000 IU D3 monthly v placebo Men and women 65-84y ISRCTN46328341 Feasibility trial. Full trial (*n* = 20,000) not funded	S:Yes Hirani 2005 PSR:Yes ASR:Yes	2012	2000	Private households	Diasorin	Hirani 2005 [93]
					Men 65-79y/80+	58 (27)/48 (24)	
					Women 65-79y/80+	49 (25)/45 (20)	
					Institutions		
					Men 65-79y/80+	40 (24)/37 (20)	
					Women 65-79y/80+	37 (18)/37 (19)	
VITAL US	N = 25,874, 5y 2000 IU/d D3 v placebo Men ≥50y, women ≥55y NCT01169259	S:Yes Looker 2002 PSR:Yes ASR:Yes	2010	1988–1994	Winter, lower latitude	Diasorin	Looker 2002 [94]
					Women 40-80y+	61.6–59.6	
					Men 40-80y+	70.6–68.7	
					Summer, higher latitude		
					Women 40-80y+	68.6–61.8	
					Men 40-80y+	78.8–69.5	
				2005–2010	Men and women	LC-MS/MS equivalent	Schleicher 2016^b^ [79]
					40-59y	60.1–68.7	
					≥60y	59.4–72.6	
				1988–1994	Men and women	LC-MS/MS equivalent	Schleicher 2016^b^ [79]
					40-59y	60.1 (58.7,61.5)	
					≥60y	58.4 (57.4,59.5)	
CAPS US	*N* = 2303, 5y 2000 IU D3 and 1500 mg calcium daily v calcium Women ≥55y NCT01052051	S:NDA PSR:NDA ASR:NDA	2009	2005–2010	Men and women	LC-MS/MS equivalent	Schleicher 2016^b^ [79]
					40-59y	60.1–68.7	
					≥60y	59.4–72.6	
					All females	60.9–69.1	

Table 3 shows that only 8 [21–28] of the 18 completed trials (44%) cited the vitamin D status of a population similar to the recruited cohort in the primary publication. One further trial [29] discussed survey data in the trial paper’s introduction, but this was not used in the grant application. Investigators from two of these trials [21, 22] had undertaken prior relevant 25OHD surveys. Four of the eight trials cited old survey data, from at least 16 years [24, 27] and 6–9 years [23, 26] before trial recruitment. A trial from Finland that studied older adults (mean age 62y) cited survey data that lacked relevance, being from the USA and from young Finnish adults (mean age 38y) [30].

Table 4 shows that all four ongoing trials with accessible documents discuss the vitamin D status of their intended trial population. One trial in Australia conducted a pilot study that included assessment of vitamin D status. The US VITAL trial which started recruitment in 2010, used NHANES III (1988–94) data in its rationale and design paper justification [31, 32].

Table 3 shows that baseline 25OHD in large completed RCTs and relevant survey 25OHD data were comparable, apart from one Norwegian trial, where one survey indicated considerably worse vitamin D status than was observed in trial participants [23]. Only one [21] of the completed trials was conducted in a population that was clearly vitamin D deficient, based on trial (mean baseline 25OHD 20 nmol/L) and survey data (mean 11–23 nmol/L). Three trials [22, 24, 29] were undertaken in populations comprised largely of participants with vitamin D insufficiency. Of the remaining 13 single country trials with baseline 25OHD or relevant survey data, five trials [23, 27, 33–35] had mean baseline 25OHD ≥40 nmol/L and four trials 25OHD ≥50 nmol/L [26, 28, 30, 36]. Four trials [25, 37–39] did not report baseline 25OHD, but surveys and data from similar RCTs suggest that baseline 25OHD in the RCT would have been ≥40 nmol/L in three of these trials [25, 37, 38]. In these 13 trials, a substantial proportion of participants would have had 25OHD ≥50 nmol/L, consistent with the IOM definition of vitamin D sufficiency [3].

Table 4 shows that, based on survey data from the relevant population, all the ongoing single country trials are likely to recruit participants in whom the mean/median baseline 25OHD will be > 40–50 nmol/L, and none describe specific strategies for recruiting participants with 25OHD < 25 nmol/L.

### Large randomised controlled trials and citation of prior systematic reviews of randomised controlled trials

We identified a relevant systematic review on vitamin D and fracture [40] published prior to trial recruitment starting for 8 completed large RCTs, and on mortality [41] for 2 RCTs, but no prior systematic reviews on colorectal adenoma or neonatal bone mineral content for two RCTs (Table 3). Thus, systematic reviews capable of informing the trial justification and design were available before trial recruitment in 10/18 (56%) of completed large RCTs. Only three [28, 29, 42] of the 10 RCTs (30%) cited such a systematic review in their primary publication. Nine trials (50%) cited systematic reviews that would have occurred after the decision had been made to undertake the trial. Four of the seven planned or ongoing trials with accessible relevant documents discuss systematic reviews in their protocols or publications: for three of the trials, the systematic reviews predate trial recruitment.

## Discussion

Our results suggest a high proportion of research waste in RCTs of vitamin D supplementation. The recent proliferation of vitamin D RCTs was accompanied by increasing baseline 25OHD concentrations and therefore a declining proportion of RCTs conducted in vitamin D deficient cohorts. Only 10% of trials were carried out in populations that would be widely accepted as vitamin D deficient, in which benefits of vitamin D supplementation still have not been unequivocally established (Table 1). Because many participants in recent trials were vitamin D sufficient, they would be unlikely to benefit from vitamin D. Further, their inclusion could have obscured potential benefits from vitamin D for those participants who were vitamin D deficient. This issue applies to RCTs with mean/median baseline 25OHD in ranges variously defined as sufficient (7% with 25OHD ≥75 nmol/L, 37% with 25OHD ≥50 nmol/L). It likely also applies to the 33% of trials with baseline 25OHD 40–49 nmol/L in which a substantial proportion of participants will have had 25OHD ≥50 nmol/L. Thus, 7–37% of trials can be considered research waste, because they were conducted in the wrong population, but this proportion is as high as 70% if a 25OHD threshold for sufficiency of 40 nmol/L was applied, based on the Institute of Medicine’s target median value.

Very importantly, research waste was prevalent in large RCTs that were designed to inform clinical practice. Only 1 such trial was carried out in a vitamin D deficient population and another 3 in populations with vitamin D insufficiency. Twelve (71%) of the remaining completed and 5 (100%) ongoing single country trials had, or are likely to have, mean baseline 25OHD > 40 nmol/L, and based on survey and other trial data, we estimate that about 50% would have 25OHD ≥50 nmol/L. Failure to incorporate key available data during protocol development may have contributed to the high prevalence of waste. Few (44%) of the large completed RCTs cited or undertook prior relevant surveys of vitamin D status in their intended trial population. Only 56% of large completed RCTs had a relevant systematic review of randomised trials published prior to trial recruitment starting and, of these, only 30% cited such a review. When systematic reviews of randomised trials were discussed, they tended to have been published after the trial had commenced or been completed. Collectively, this suggests that these large, costly RCTs were not optimally designed to address the question of benefits of vitamin D supplements.

An important strength of this study assessing research waste is that we analysed the complete set of RCTs of vitamin D published over 30 years. The results from this single research area might not apply to other research fields, and waste may be more prevalent in mature as opposed to emerging areas of research. In assessing whether trials cited 25OHD surveys or relevant systematic reviews, we examined primary publications and protocols where available. Our results may have changed if we were able to examine grant applications and trial protocols, but protocols were often not available, and we had access to only one grant application [29]. Early 25OHD assays tended to overestimate 25OHD- we used 25OHD concentrations corrected for these overestimates. The corrected values are approximations, but nevertheless lower than the original values in the relevant trials and surveys, and therefore the proportions of participants with vitamin D deficiency in our analyses are higher than in the original publications. Very few RCTs reported the season when 25OHD measurements were obtained. Although seasonal changes in 25OHD will occur in all treatment arms, it is possible that seasonal effects of 25OHD might confound some trial results. A limitation of this study is that the literature search was conducted in December 2015.

The implications of this research are that the current body of RCTs of vitamin D with clinical endpoints, including large RCTs with ≥1000 participants, is largely conducted in populations that are not vitamin D deficient. Recent, large systematic reviews of these RCTs report no benefits of vitamin D [8–11]. In trials included in these meta-analyses reporting 25OHD, 72–75% had baseline 25OHD < 50 nmol/L [10, 43], consistent with Fig. 1d showing that the majority of trials prior to 2011 had baseline 25OHD < 50 nmol/L. Thus, it is reasonable to conclude that current evidence is sufficient to exclude benefits from vitamin D supplementation for unselected community-dwelling individuals with 25OHD > 30–40 nmol/L. Relatively few trials, including only 5003 participants (Table 1), have been carried out in populations with lower baseline 25OHD and their results are inconsistent, with only 33% of such trials reporting beneficial results from vitamin D. Subgroup analyses of participants with lower 25OHD at baseline were frequently undertaken but their results were invariably similar to the results of the main analyses for the primary endpoint, even when the subgroup was restricted to people with 25OHD ≤25 nmol/L. Therefore, it is uncertain whether vitamin D supplementation benefits people with clearly low 25OHD. Based on data from relevant 25OHD surveys, the large RCTs currently underway will not test the effects of vitamin D supplementation in deficient populations and therefore are unlikely to address this knowledge gap. Instead of continuing to spend resources on trials in vitamin D sufficient populations, investigators should focus on vitamin D deficient populations. Food fortification policies [15, 16], together with independent action by food manufacturers and new advice on supplementation [5], make it even less likely that future trials in deficient populations will be possible.

Our analyses suggest that up to 70% of RCTs with clinical endpoints, 71% of large (*N* ≥ 1000) completed RCTs, and 100% of ongoing large RCTs could be considered research waste because they studied cohorts with a high proportion of vitamin D sufficiency. In our companion paper [2], we reported that 69% of RCTs of vitamin D conducted since 2005 with skeletal endpoints of bone mineral density or fracture were research waste because they lacked novelty or did not add to existing clinical knowledge. Taken together, these findings support the very high proportions (> 85%) for research waste estimated by Chalmers and Glasziou [1].

## Conclusions

We identified a very high proportion of research waste in RCTs of vitamin D with clinical endpoints. Few RCTs were carried out in vitamin D deficient populations most likely to benefit from vitamin D supplementation, and conversely most RCTs were carried out in populations unlikely to benefit from supplementation. Few large RCTs appeared to consider systematic reviews in their design. Ongoing large RCTs share the same weaknesses of previous trials. Strategies to improve the design of RCTs should be introduced and studied to determine whether they can reduce research waste.

## Additional file

Additional file 1: **Table S1.** Searches of Pubmed undertaken in December 2015. **Table S2.** 38 Systematic reviews identified in Pubmed search. **Table S3.** Characteristics of 137 randomised controlled trials of vitamin D supplements with clinical endpoints reported in abstract. **Table S4.** Large completed randomised controlled trials of vitamin D supplements with relevant prior 25-hydroxyvitamin D surveys. **Table S5.** Large ongoing randomised controlled trials of vitamin D supplements with relevant prior 25-hydroxyvitamin D surveys. **Figure S1.** flow of studies. References. (DOCX 313 kb)

## Abbreviations

25OHD: 25-hydroxyvitamin D
CBP: Competitive binding protein
RCT: Randomised controlled trial
