# Supplementary material for: Assessment of research waste part 2: wrong study populations- an exemplar of baseline vitamin D status of participants in trials of vitamin D supplementation
Source: BMC Med Res Methodol. 2018 Oct 3;18:101. doi: 10.1186/s12874-018-0555-1 (PMC6171194; doi:10.1186/s12874-018-0555-1)
Supplement: Supplementary file 1 — Table S1. Searches of Pubmed undertaken in December 2015. Table S2. 38 Systematic reviews identified in Pubmed search. Table S3. Characteristics of 137 randomised controlled trials of vitamin D supplements with clinical endpoints reported in abstract. Table S4. Large completed randomised controlled trials of vitamin D supplements with relevant prior 25-hydroxyvitamin D surveys. Table S5. Large ongoing randomised controlled trials of vitamin D supplements with relevant prior 25-hydroxyvitamin D surveys. Figure S1. flow of studies. References. (DOCX 313 kb) [file 12874_2018_555_MOESM1_ESM.docx]

**Additional file 1**

**Table S1: Searches of Pubmed undertaken in December 2015**

| **Database** | **Search Terms** | **Citations** |
| --- | --- | --- |
| Pubmed | Vitamin D with clinical trials filter | 4018 |
| Pubmed | Within title: (“Vitamin D” or “cholecalciferol” or “colecalciferol” or “ergocalciferol” or “calciferol”) and (random* or “trial”) | 631 |
| Pubmed | Vitamin D, publication date after 1/1/2015 | 634 |
| Pubmed | Systematic reviews or Meta-analyses of randomized controlled trials of vitamin D with clinical endpoints from Table 3 and major surrogate endpoints. We included the latest 2 publications on each topic but included any review published in 2014-5 | 38 |

**Table S2: 38 Systematic reviews identified in Pubmed search**

| **Subject** | **Reference** |
| --- | --- |
| Rheumatoid Arthritis, Systemic lupus erythematosus | Antico A, Tampoia M, Tozzoli R, Bizzaro N. Can supplementation with vitamin D reduce the risk or modify the course of autoimmune diseases? A systematic review of the literature. Autoimmun Rev. 2012;12(2):127-136. |
| Mood; Diabetes; Cancer; Multiple sclerosis; | Autier P, Boniol M, Pizot C, Mullie P. Vitamin D status and ill health: a systematic review. Lancet Diabetes Endocrinol. 2014;2(1):76-89. |
| Fracture; Mortality | Avenell A, Mak JC, O'Connell D. Vitamin D and vitamin D analogues for preventing fractures in post-menopausal women and older men. Cochrane Database Syst Rev. 2014;4:CD000227. |
| Muscle strength | Beaudart C, Buckinx F, Rabenda V, et al. The effects of vitamin D on skeletal muscle strength, muscle mass, and muscle power: a systematic review and meta-analysis of randomized controlled trials. J Clin Endocrinol Metab. 2014;99(11):4336-4345. |
| Respiratory tract infection | Bergman P, Lindh AU, Bjorkhem-Bergman L, Lindh JD. Vitamin D and Respiratory Tract Infections: A Systematic Review and Meta-Analysis of Randomized Controlled Trials. PLoS One. 2013;8(6):e65835. |
| Blood pressure | Beveridge LA, Struthers AD, Khan F, et al. Effect of Vitamin D Supplementation on Blood Pressure: A Systematic Review and Meta-analysis Incorporating Individual Patient Data. JAMA Intern Med. 2015;175(5):745-754. |
| Cancer | Bjelakovic G, Gluud LL, Nikolova D, et al. Vitamin D supplementation for prevention of cancer in adults. Cochrane Database Syst Rev. 2014;6:CD007469. |
| Mortality | Bjelakovic G, Gluud LL, Nikolova D, et al. Vitamin D supplementation for prevention of mortality in adults. Cochrane Database Syst Rev. 2014;1:CD007470. |
| Fracture | Bolland MJ, Grey A. A case study of discordant overlapping meta-analyses: vitamin d supplements and fracture. PLoS One. 2014;9(12):e115934. |
| Fracture, Cardiovascular events; Cancer, Mortality | Bolland MJ, Grey A, Gamble GD, Reid IR. The effect of vitamin D supplementation on skeletal, vascular, or cancer outcomes: a trial sequential meta-analysis. Lancet Diabetes Endocrinol. 2014;2(4):307-320. |
| Falls | Bolland MJ, Grey A, Gamble GD, Reid IR. Vitamin D supplementation and falls: a trial sequential meta-analysis. Lancet Diabetes Endocrinol. 2014;2(7):573-580. |
| Falls | Bolland MJ, Grey A, Reid IR. Differences in overlapping meta-analyses of vitamin d supplements and falls. J Clin Endocrinol Metab. 2014;99(11):4265-4272. |
| Colorectal adenoma | Carroll C, Cooper K, Papaioannou D, Hind D, Pilgrim H, Tappenden P. Supplemental calcium in the chemoprevention of colorectal cancer: a systematic review and meta-analysis. Clin Ther. 2010;32(5):789-803. |
| Weight | Chandler PD, Wang L, Zhang X, et al. Effect of vitamin D supplementation alone or with calcium on adiposity measures: a systematic review and meta-analysis of randomized controlled trials. Nutr Rev. 2015;73(9):577-593. |
| Mortality | Chowdhury R, Kunutsor S, Vitezova A, et al. Vitamin D and risk of cause specific death: systematic review and meta-analysis of observational cohort and randomised intervention studies. BMJ. 2014;348:g1903. |
| Depression | Gowda U, Mutowo MP, Smith BJ, Wluka AE, Renzaho AM. Vitamin D supplementation to reduce depression in adults: meta-analysis of randomized controlled trials. Nutrition. 2015;31(3):421-429. |
| Pregnancy | Harvey NC, Holroyd C, Ntani G, et al. Vitamin D supplementation in pregnancy: a systematic review. Health Technol Assess. 2014;18(45):1-190. |
| General health | Hoffmann MR, Senior PA, Mager DR. Vitamin D supplementation and health-related quality of life: a systematic review of the literature. J Acad Nutr Diet. 2015;115(3):406-418. |
| Glycaemia | Jamka M, Wozniewicz M, Jeszka J, Mardas M, Bogdanski P, Stelmach-Mardas M. The effect of vitamin D supplementation on insulin and glucose metabolism in overweight and obese individuals: systematic review with meta-analysis. Sci Rep. 2015;5:16142. |
| Cancer | Keum N, Giovannucci E. Vitamin D supplements and cancer incidence and mortality: a meta-analysis. Br J Cancer. 2014;111(5):976-980. |
| Depression | Li G, Mbuagbaw L, Samaan Z, et al. Efficacy of vitamin D supplementation in depression in adults: a systematic review. J Clin Endocrinol Metab. 2014;99(3):757-767. |
| Lipids, weight, blood pressure | Manousopoulou A, Al-Daghri NM, Garbis SD, Chrousos GP. Vitamin D and cardiovascular risk among adults with obesity: a systematic review and meta-analysis. Eur J Clin Invest. 2015;45(10):1113-1126. |
| Respiratory tract infection | Mao S, Huang S. Vitamin D supplementation and risk of respiratory tract infections: a meta-analysis of randomized controlled trials. Scand J Infect Dis. 2013;45(9):696-702. |
| Crohn’s disease | Nicholson I, Dalzell AM, El-Matary W. Vitamin D as a therapy for colitis: a systematic review. J Crohns Colitis. 2012;6(4):405-411. |
| Pregnancy | Perez-Lopez FR, Pasupuleti V, Mezones-Holguin E, et al. Effect of vitamin D supplementation during pregnancy on maternal and neonatal outcomes: a systematic review and meta-analysis of randomized controlled trials. Fertil Steril. 2015;103(5):1278-1288 e1274. |
| Multiple sclerosis | Pozuelo-Moyano B, Benito-Leon J, Mitchell AJ, Hernandez-Gallego J. A systematic review of randomized, double-blind, placebo-controlled trials examining the clinical efficacy of vitamin D in multiple sclerosis. Neuroepidemiology. 2013;40(3):147-153. |
| Bone density | Reid IR, Bolland MJ, Grey A. Effects of vitamin D supplements on bone mineral density: a systematic review and meta-analysis. Lancet. 2014;383(9912):146-155. |
| Systemic lupus erythematosus | Sakthiswary R, Raymond AA. The clinical significance of vitamin D in systemic lupus erythematosus: a systematic review. PLoS One. 2013;8(1):e55275. |
| Glycaemia | Sarathy H, Pramanik V, Kahn J, et al. The effects of short-term vitamin D supplementation on glucose metabolism in dialysis patients: a systematic review and meta-analysis. Int Urol Nephrol. 2015;47(3):537-549. |
| Glycaemia; Diabetes | Seida JC, Mitri J, Colmers IN, et al. Clinical review: Effect of vitamin D3 supplementation on improving glucose homeostasis and preventing diabetes: a systematic review and meta-analysis. J Clin Endocrinol Metab. 2014;99(10):3551-3560. |
| Mood | Shaffer JA, Edmondson D, Wasson LT, et al. Vitamin D supplementation for depressive symptoms: a systematic review and meta-analysis of randomized controlled trials. Psychosom Med. 2014;76(3):190-196. |
| Bone density | Silk LN, Greene DA, Baker MK. The Effect of Calcium or Calcium and Vitamin D Supplementation on Bone Mineral Density in Healthy Males: A Systematic Review and Meta-Analysis. Int J Sport Nutr Exerc Metab. 2015;25(5):510-524. |
| Endothelial function | Stojanovic M, Radenkovic M. Vitamin D versus placebo in improvement of endothelial dysfunction: a meta-analysis of randomized clinical trials. Cardiovasc Ther. 2015;33(3):145-154. |
| Tuberculosis | Sutaria N, Liu CT, Chen TC. Vitamin D Status, Receptor Gene Polymorphisms, and Supplementation on Tuberculosis: A Systematic Review of Case-Control Studies and Randomized Controlled Trials. J Clin Transl Endocrinol. 2014;1(4):151-160. |
| Bone density | Tai V, Leung W, Grey A, Reid IR, Bolland MJ. Calcium intake and bone mineral density: systematic review and meta-analysis. BMJ. 2015;351:h4183. |
| Mortality, Fracture, Falls | Theodoratou E, Tzoulaki I, Zgaga L, Ioannidis JP. Vitamin D and multiple health outcomes: umbrella review of systematic reviews and meta-analyses of observational studies and randomised trials. BMJ. 2014;348:g2035. |
| Muscle strength | Tomlinson PB, Joseph C, Angioi M. Effects of vitamin D supplementation on upper and lower body muscle strength levels in healthy individuals. A systematic review with meta-analysis. J Sci Med Sport. 2015;18(5):575-580. |
| Tuberculosis | Xia J, Shi L, Zhao L, Xu F. Impact of vitamin D supplementation on the outcome of tuberculosis treatment: a systematic review and meta-analysis of randomized controlled trials. Chin Med J (Engl). 2014;127(17):3127-3134. |

No systematic reviews of randomised controlled trials of vitamin D were identified for Asthma, Chronic obstructive pulmonary disease, Pain, Dermatitis, ICU admissions, Seizure, Raynauds, Gingivitis, Osteoarthritis, Migraine, Uraemia, Chillblains, Alzheimer’s disease, Parkinson’s disease, Leg ulcer, Bacterial vaginosis, Vascular fistula, Infertility, Vaginal atrophy, Chronic fatigue, and Hepatitis.

**Table S3: Characteristics of 137 randomised controlled trials of vitamin D supplements with clinical endpoints reported in abstract.**

| **Study** | **Clinical endpoint** | **Location** | **Inclusion**  **criterion**  **based on 25OHD** | **Study**  **size**  **(N)** | **25OHD**  **levels^a^** | **25OHD**  **Assay** | **Mean/**  **Median**  **25OHD**  **(nmol/L)** |
| --- | --- | --- | --- | --- | --- | --- | --- |
| Abou-Raya 2013^1^ | SLE disease activity | Egypt | <30 ng/ml | 267 | All | Liaison | 50 |
| Aflatoonian 2014^2^ | Pregnancy rate | Iran | <30 ng/ml | 114 | All | ELISA | 37 |
| Aloia 2007^3^ | URTI | USA | No | 208 | All | Diasorin | 46 |
| Amestejani 2012^4^ | Atopic dermatitis | Iran | No | 60 | All | Biosource | 24 |
| Aminmansour 2012^5^ | Head injury score | Iran | No | 40 | ND | ND |  |
| Amrein 2014^6^ | ICU stay | Austria | ≤20 ng/ml | 492 | All | IDS | 33 |
| Andreoli 2015^7^ | SLE disease activity | Italy | No | 34 | All | Liaison | 80 |
| Arvold 2009^8^ | Musculoskeletal symptoms | USA | 10-25 ng/ml | 100 | All | LCMS/MS | 45 |
| Asadi 2014^9^ | Pregnancy rates | Iran | <100 ng/ml | 110 | All | NS | 31 |
| Asemi 2015^10^ | Pregnancy outcomes | Iran | No | 50 | All | IDS | 50 |
| Baron 2015^11^ | Colorectal adenoma | USA | 12-90 ng/ml | 2259 | All | IDS | 61 |
| Bergman 2012^12^ | RTI symptoms | Sweden | No | 140 | All | NS | 49 |
| Bhan 2015^13^ | All-cause mortality | USA | ≤32 ng/ml | 105 | All | LCMS/MS | 22 |
| Bischoff 2003^14^ | Falls | Switzerland | No | 122 | All | Nicholls | 23 |
| Bischoff-Ferrari 2010^15^ | Falls | Switzerland | No | 173 | All | Diasorin | 32 |
| Bjorkman 2008^16^ | Pain | Finland | No | 202 | All | HPLC | 56 |
| Broe 2007^17^ | Falls | USA | No | 124 | All | NS | 49 |
| Brohult 1973^18^ | RA disease activity | Sweden | No | 49 | ND | ND |  |
| Brooke 1980^19^ | Newborn outcomes | UK | No | 126 | All | CPB | 11 |
| Burkiewicz 2012^20^ | Leg ulcer healing | Brazil | <30 ng/ml | 21 | All | Liaison | 38 |
| Burleigh 2007^21^ | Falls | Scotland | No | 205 | Subg | Nicholl | 44 |
| Burton 2010^22^ | MS relapse | Canada | <150 nmol/L | 49 | All | NS | 78 |
| Cangussu 2015^23^ | Falls | Brazil | No | 160 | All | NS | 40 |
| Castro 2014^24^ | Asthma | USA | <30 ng/ml | 408 | All | Liaison | 47 |
| Catalano 2012^25^ | Pain | Italy | No | 60 | All | HPLC | 80 |
| Chapuy 1994^26^ | Fracture | France | No | 3270 | Subg | CBP | 20 |
| Chapuy 2002^27^ | Fracture | France | No | 583 | All | Incstar | 22 |
| Christiansen 1974^28^ | Epilepsy | Denmark | No | 23 | ND | ND |  |
| Daley 2015^29^ | TB sputum culture | India | No | 247 | All | Roche Cobas | 63 |
| Davidson 2013^30^ | Diabetes | USA | <30 ng/ml | 109 | All | LCMS/MS | 55 |
| Dawson-Hughes 1987^31^ | Fracture | USA | No | 389 | All | CBP | 40 |
| de Gruijl 2012^32^ | URTI | Holland | No | 70 | All | IDS | 60 |
| Dehghan 2014^33^ | RA disease activity | Iran | "Normal" | 80 | ND | ND |  |
| Deng 2014^34^ | Pregnancy rate | China | No | 86 | ND | ND |  |
| Derakhshandi 2013^35^ | MS incidence | Iran | <30 ng/ml | 30 | All | Diasorin | 38 |
| Dutta 2014^36^ | Diabetes | India | <30 ng/ml | 125 | All | NS | 44 |
| Etemadifar 2015^37^ | MS relapse | Iran | <20 ng/ml | 43 | All | Diasorin | 43 |
| Firouzabadi 2012^38^ | Pregnancy rate | Iran | No | 100 | All | RIA | 34 |
| Flicker 2005^39^ | Falls | Australia | 25-90 nmol/L | 625 | All | Incstar | NS |
| Frandsen 2014^40^ | Seasonal affective disorder | Denmark | 10-160 nmol/L | 43 | All | IDS | NS |
| Gendelman 2015^41^ | Pain | Israel | No | 74 | All | Liaison | 59 |
| Glendenning 2012^42^ | Falls | Australia | No | 686 | Subg | Liaison | 66 |
| Golan 2013^43^ | MS disease score/relapse | Israel | <75 nmol/L | 45 | All | Liaison | 48 |
| Goldring 2013^44^ | Childhood wheeze | UK | No | 180 | All | NS | 26 |
| Goodall 2014^45^ | URTI | Canada | No | 600 | ND | ND |  |
| Grant 2005^46^ | Fracture | UK | No | 5292 | Subg | HPLC | 38 |
| Grant 2015^47^ | URTI | New Zealand | No | 250 | All | LCMS/MS | 56 |
| Grossmann 2012^48^ | Cystic fibrosis outcomes | USA | 5-75 ng/ml | 30 | All | IDS | 74 |
| Hansen 2014^49^ | RA disease activity | USA | 6.1-24.9 ng/ml | 22 | All | HPLC | 58 |
| Harwood 2004^50^ | Falls | UK | No | 150 | All | Incstar | 29 |
| Hata 2014^51^ | Atopic dermatitis | USA | No | 76 | All | NS | 74 |
| Helou 2013^52^ | Raynauds | France | <30 ng/ml | 42 | All | NS | 53 |
| Hiremath 2013^53^ | Gingivitis | India | 20-65 ng/ml | 96 | All | Immunotek | 63 |
| Hollis 2011^54^ | Pregnancy outcomes | USA | No | 502 | All | HPLC | 59 |
| Hossain 2014^55^ | Pregnancy outcomes | Pakistan | No | 200 | All | Immunoassay | 13 |
| Houston 2015^56^ | Falls | USA | No | 68 | All | NS | 52 |
| Inkovaara 1983^57^ | Fracture | Finland | No | 327 | ND | ND |  |
| Jackson 2006^58^ | Fracture | USA | No | 36282 | Subg | Liaison | 48 |
| Jorde 2008^59^ | Depression symptoms | Norway | No | 445 | All | Roche | 52 |
| Jorgensen 2010^60^ | Crohn's relapse | Denmark | No | 94 | All | NS | 73 |
| Kampman 2012^61^ | MS relapse | Norway | No | 71 | All | LCMS/MS | 56 |
| Karamali 2015^62^ | Pregnancy outcomes | Iran | No | 60 | All | IDS | 48 |
| Khajehei 2010^63^ | General health | Iran | No | 120 | ND | ND |  |
| Khoraminya 2013^64^ | Depression scores | Iran | No | 42 | All | ELISA | 58 |
| Kjaergaard 2012^65^ | Depression scores | Norway | <55 nmol/L | 243 | All | LCMS/MS | 48 |
| Knutsen 2014^66^ | Pain | Norway | No | 251 | All | LCMS/MS | 27 |
| Komulainen 1998^67^ | Fracture | Finland | No | 464 | ND | ND |  |
| Kota 2011^68^ | TB sputum smear conversion | India | <20 ng/ml | 30 | All | NS | 30 |
| Kuchay 2015^69^ | Diabetes | India | No | 137 | All | Diasorin | 48 |
| Laaksi 2010^70^ | Respiratory tract infection | Finland | No | 160 | Subg | IDS | 76 |
| Lappe 2007^71^ | Cancer | USA | No | 891 | All | IDS | 72 |
| Lappe 2008^72^ | Stress fracture | USA | No | 5201 | ND | ND |  |
| Larsen 2004^73^ | Fracture | Denmark | No | 5063 | Subg | Diasorin | 36 |
| Lasco 2012^74^ | Dysmenorrhoea | Italy | <45 ng/ml | 40 | All | NS | 72 |
| Latham 2003^75^ | Falls | New Zealand | No | 243 | All | Diasorin | 43 |
| Law 2006^76^ | Fracture | UK | No | 3717 | Subg | IDS | 47 |
| Lehouck 2012^77^ | COPD | Belgium | No | 182 | All | Diasorin | 50 |
| Li-Ng 2009^78^ | URTI | USA | No | 162 | All | Diasorin | 64 |
| Lips 1996^79^ | Fracture | Holland | No | 2578 | Subg | HPLC | 27 |
| Lopez-Torres  Hidalgo 2014^80^ | Falls | Spain | <10 ng/ml | 398 | All | NS | 83 |
| Lyons 2007^81^ | Fracture | UK | No | 3440 | ND | ND |  |
| Martineau 2011^82^ | TB sputum smear conversion | UK | No | 126 | All | LCMS/MS | 21 |
| Martineau 2015^83^ | Asthma | UK | No | 250 | All | LCMS/MS | 50 |
| Martineau 2015^84^ | URTI | UK | No | 240 | All | LCMS/MS | 43 |
| Martineau 2015^85^ | COPD | UK | No | 240 | All | LCMS/MS | 46 |
| Marya 1987^86^ | Toxaemia of pregnancy | India | No | 400 | ND | ND |  |
| McAlindon 2013^87^ | Knee pain of OA | USA | No | 146 | All | LCMS/MS | 56 |
| Meyer 2002^88^ | Fracture | Norway | No | 1144 | Subg | HPLC | 49 |
| Miskulin 2015^89^ | Hospitalisation | USA | <30 ng/ml | 276 | All | Liaison | 41 |
| Mosayebi 2011^90^ | MS scores | Iran | No | 59 | All | IDS | 25 |
| Mottaghi 2015^91^ | Migraine | Iran | No | 65 | All | ELISA | 45 |
| Mozaffari-Khosravi  2013^92^ | Depression scores | Iran | < 40 nmol/L | 120 | All | IDS | 23 |
| Murdoch 2012^93^ | URTI | New Zealand | No | 322 | All | LCMS/MS | 71 |
| Nair 2015^94^ | Mortality | Australia | No | 50 | All | LCMS/MS | 47 |
| Nursyam 2006^95^ | TB sputum smear conversion | Indonesia | No | 67 | ND | ND |  |
| Pfeifer 2000^96^ | Falls | Germany | <50 nmol/L | 148 | All | Nicholls | 19 |
| Pfeifer 2009^97^ | Falls | Germany | <78 nmol/L | 242 | All | IDS | 55 |
| Porthouse 2005^98^ | Fracture | UK | No | 3314 | ND | ND |  |
| Prince 2008^99^ | Falls | Australia | <24 ng/ml | 302 | All | Diasorin | 45 |
| Punthakee 2012^100^ | Cancer or All-cause mortality | Multinational | No | 1221 | ND | ND |  |
| Raftery 2015^101^ | Crohn's disease activity | Ireland | No | 27 | All | LCMS/MS | 60 |
| Ralph 2013^102^ | TB sputum culture | Indonesia | No | 200 | ND | ND |  |
| Rashidi 2009^103^ | Pregnancy rate | Iran | No | 40 | ND | ND |  |
| Rastelli 2011^104^ | Musculoskeletal pain | USA | 10-29 ng/ml | 60 | All | Liaison | 56 |
| Rizzoli 2014^105^ | Falls | Multinational | >22.5 nmol/L | 518 | All | Diasorin | 44 |
| Rorie 2014^106^ | Chronic urticaria | USA | No | 42 | All | LCMS/MS | 82 |
| Roth 2013^107^ | Pregnancy outcomes | Bangladesh | No | 160 | All | LCMS/MS | 45 |
| Sablok 2015^108^ | Pregnancy outcomes | India | No | 180 | All | ELISA | 33 |
| Sakalli 2012^109^ | Pain | Turkey | No | 120 | All | IDS | 52 |
| Salahuddin 2013^110^ | TB chest x-ray improvement | Saudi Arabia | No | 259 | All | Roche | 54 |
| Salesi 2012^111^ | RA disease activity | Iran | No | 98 | All | NS | 100 |
| Salovaara 2010^112^ | Fracture | Finland | No | 3432 | Subg | Diasorin | 50 |
| Sanders 2010^113^ | Fracture | Australia | No | 2258 | Subg | Diasorin | 50 |
| Sandoughi 2015^114^ | Back pain | Iran | No | 53 | All | ELISA | 47 |
| Sanghi 2013^115^ | Knee pain of OA | India | <50 nmol/L | 106 | All | IDS | 37 |
| Schleithoff 2006^116^ | Mortality | Germany | No | 123 | All | Diasorin | 37 |
| Schreuder 2012^117^ | Pain | Holland | <50 nmol/L | 84 | All | Diasorin | 20 |
| Sepehrmanesh 2015^118^ | Depression scores | Iran | No | 40 | All | IDS | 29 |
| Shirazian 2013^119^ | Uraemic pruritus | USA | No | 50 | All | NS | 44 |
| Smith 2007^120^ | Fracture | UK | No | 9440 | Subg | Nicholls | 43 |
| Souwer 2009^121^ | Chill blains | Holland | No | 33 | ND | ND |  |
| Stein 2011^122^ | MS disease score/relapse | Australia | No | 23 | All | Diasorin | 56 |
| Stein 2011^123^ | Alzheimer disease score | Australia | <90 nmol/L | 32 | All | Diasorin | 62 |
| Suzuki 2013^124^ | Parkinson's disease score | Japan | No | 114 | All | Diasorin | 55 |
| Tran 2014^125^ | Antibiotic prescriptions | Australia | No | 644 | All | Liaison | 42 |
| Trivedi 2003^126^ | Fracture | UK | No | 2686 | ND | ND |  |
| Tukvadze 2015^127^ | TB sputum culture | USA | No | 199 | All | LCMS/MS | 37 |
| Turner 2014^128^ | Bacterial vaginosis | USA | No | 118 | All | Liaison | 41 |
| Uusi-Rasi 2015^129^ | Falls | Finland | No | 409 | All | IDS | 67 |
| Vieth 2004^130^ | Wellbeing | Canada | <51/<61nmol/L | 130 | All | Diasorin | 44 |
| Wagner 2013^131^ | Pregnancy outcomes | USA | No | 257 | All | Diasorin | 57 |
| Warner 2008^132^ | Pain | USA | 9-20 ng/ml | 50 | All | LCMS/MS | 41 |
| Wasse 2014^133^ | AV fistula maturation | USA | No | 52 | All | Liaison | 39 |
| Wejse 2009^134^ | TB score | Guinea-Basseau | No | 367 | All | LCMS/MS | 78 |
| Wepner 2014^135^ | Pain | Austria | <80 nmol/L | 30 | All | NS | 50 |
| Witham 2015^136^ | Chronic fatigue score | UK | <75 nmol/L | 50 | All | IDS | 46 |
| Wood 2014^137^ | Falls | UK | No | 305 | All | LCMS/MS | 34 |

^a^ Whether baseline 25OHD measurements were performed, and if so, in all participants or subgroups.

Abbreviations: 25OHD- 25-hydroxyvitamin D; SLE –Systemic lupus erythematosus; URTI- upper respiratory tract infection; ICU- intensive care unit; TB- tuberculosis; RA rheumatoid arthritis; MS- multiple sclerosis; AV- arteriovenous; NS- not stated; ND- not done; Subg- subgroup of participants; ELISA- enzyme-linked immunosorbent assay; IDS - Immunodiagnostic systems: LCMS/MS- liquid chromatography tandem mass-spectrometry; HPLC - high performance liquid chromatography; CPB- competitive binding protein; RIA- radioimmunoassay

**Table S4: Large completed randomised controlled trials of vitamin D supplements with relevant prior 25-hydroxyvitamin D surveys**

| **Study** | **Reference for relevant survey** |
| --- | --- |
| Chapuy 1994^26^ | Chapuy MC, Chapuy P, Meunier PJ. Calcium and vitamin D supplements: effects on calcium metabolism in elderly people. Am J Clin Nutr 1987;46:324-8. |
| Lips 1996^79^ | Lips P, van Ginkel FC, Jongen MJ, Rubertus F, van der Vijgh WJ, Netelenbos JC. Determinants of vitamin D status in patients with hip fracture and in elderly control subjects. Am J Clin Nutr 1987;46:1005-10  Lowik MR, Schrijver J, Odink J, van den Berg H, Wedel M, Hermus RJ. Nutrition and aging: nutritional status of “apparently healthy” elderly (Dutch nutrition surveillance system). J Am Coll Nutr 1990;9:18-27 |
|  | Lips P, Wiersinga A, van Ginkel FC, Jongen MJ, Netelenbos JC, Hackeng WH, Delmas PD, van der Vijgh WJ. The effect of vitamin D supplementation on vitamin D status and parathyroid function in elderly subjects. J Clin Endocrinol Metab 1988;67:644-50 |
| Meyer 2002^88^ | Mowe M, Bohmer T, Haug E. Vitamin D-mangel hos eldre sykehusinnlagte og hjemmeboende i Oslo. Tidsskr Nor Laegeforen 1998;118:3929-31  Nes M, Lund-Larsen K, Trygg K, Hoivik HO, Pedersen JI. Nutrition and the elderly in Europe: low prevalence of obesity and biochemical deficiencies in Norwegian subjects. Age Nutr 1993;4:72-81 |
| Trivedi 2003^126^ | Finch S, Doyle W, Lowe C, Bates CJ, Prentice A, Smithers G, Clarke PC. National diet and nutrition survey: people aged 65 years and over. London: The Stationery Office; 1998. |
| Larsen 2004^73^ | Lund B, Sorensen OH. Measurement of 25-hydroxyvitamin D in serum and its relation to sunshine, age and vitamin D intake in the Danish population. Scand J Clin Lab Invest 1979;39:23-30  van der Wielen RP, Lowik MR, van den Berg H, de Groot LC, Haller J, Moreiras O, van Staveren WA. Serum vitamin D concentrations among elderly people in Europe. Lancet 1995;346:207-10 |
| Grant 2005^46^ | Finch S, Doyle W, Lowe C, Bates CJ, Prentice A, Smithers G, Clarke PC. National diet and nutrition survey: people aged 65 years and over. London: The Stationery Office; 1998. |
| Porthouse 2005^98^ | Finch S, Doyle W, Lowe C, Bates CJ, Prentice A, Smithers G, Clarke PC. National diet and nutrition survey: people aged 65 years and over. London: The Stationery Office; 1998. |
| Jackson 2006^58^ | ^a^Schleicher RL, Sternberg MR, Lacher DA, Sempos CT, Looker AC, Durazo-Arvizu RA, Yetley EA, Chaudhary-Webb M, Maw KL, Pfeiffer CM, Johnson CL. The vitamin D status of the US population from 1988 to 2010 using standardized serum concentrations of 25-hydroxyvitamin D shows recent modest increases. Am J Clin Nutr 2016;104:454-61. |
| Law 2006^76^ | Finch S, Doyle W, Lowe C, Bates CJ, Prentice A, Smithers G, Clarke PC. National diet and nutrition survey: people aged 65 years and over. London: The Stationery Office; 1998. |
| Lyons 2007^81^ | Finch S, Doyle W, Lowe C, Bates CJ, Prentice A, Smithers G, Clarke PC. National diet and nutrition survey: people aged 65 years and over. London: The Stationery Office; 1998. |
| Smith 2007^120^ | Finch S, Doyle W, Lowe C, Bates CJ, Prentice A, Smithers G, Clarke PC. National diet and nutrition survey: people aged 65 years and over. London: The Stationery Office; 1998. |
| Lappe 2008^72^ | Gordon CM, DePeter KC, Feldman HA, Grace E, Emans SJ. Prevalence of vitamin D deficiency among healthy adolescents. Arch Pediatr Adolesc Med 2004;158:531-7. |
|  | ^a^Schleicher RL, Sternberg MR, Lacher DA, Sempos CT, Looker AC, Durazo-Arvizu RA, Yetley EA, Chaudhary-Webb M, Maw KL, Pfeiffer CM, Johnson CL. The vitamin D status of the US population from 1988 to 2010 using standardized serum concentrations of 25-hydroxyvitamin D shows recent modest increases. Am J Clin Nutr 2016;104:454-61. |
| Salovaara 2010^112^ | Kauppi M, Impivaara O, Maki J, Heliovaara M, Marniemi J, Montonen J, Jula A. Vitamin D status and common risk factors for bone fragility as determinants of quantitative ultrasound variables in a nationally representative population sample. Bone 2009;45:119-24 |
| Sanders 2010^113^ | Pasco JA, Henry MJ, Nicholson GC, Sanders KM, Kotowicz MA. Vitamin D status of women in the Geelong Osteoporosis Study: association with diet and casual exposure to sunlight. Med J Aust 2001;175:401-5 |
| Punthakee 2012^100^ |  |
| Baron 2015^11^ | ^a^Schleicher RL, Sternberg MR, Lacher DA, Sempos CT, Looker AC, Durazo-Arvizu RA, Yetley EA, Chaudhary-Webb M, Maw KL, Pfeiffer CM, Johnson CL. The vitamin D status of the US population from 1988 to 2010 using standardized serum concentrations of 25-hydroxyvitamin D shows recent modest increases. Am J Clin Nutr 2016;104:454-61. |
| Cooper 2016^138^ | Javaid MK, Crozier SR, Harvey NC, Gale CR, Dennison EM, Boucher BJ, Arden NK, Godfrey KM, Cooper C, and the Princess Anne Hospital Study Group. Maternal vitamin D status during pregnancy and childhood bone mass at age 9 years: longitudinal study. Lancet 2006;367:36-43. |
|  | National Diet and Nutrition Survey. Results from years 1, 2, 3 and 4 (combined) of the Rolling Programme (2008/2009 – 2011/2012). London: Public Health London; 2014. Available from:  https://www.gov.uk/government/uploads/system/uploads/attachment_data/file/310995/NDNS_Y1_to_4_UK_report.pdf |
| Scragg 2017^139^ | P: Scragg R, Waayer D, Stewart AW, Lawes CM, Top L, Murphy J, Khaw KT, Camargo CA. The Vitamin D Assessment (ViDA) Study: design of a randomized controlled trial of vitamin D supplementation for the prevention of cardiovascular disease, acute respiratory infection, falls and non-vertebral fractures. J Steroid Biochem Molecul Biol [doi:10.1016/j.jsbmb.2015.09.010](http://dx.doi.org/10.1016/j.jsbmb.2015.09.010) |
|  | S: Rockell JE, Skeaff CM, Williams SM, Green TJ. Serum 25-hydroxyvitamin D concentrations of New Zealanders aged 15 years and older. Osteoporosis Int 2006;17:1382-9. |
|  | S:Ministry of Health. 2012. Vitamin D status of New Zealand adults: Findings from the 2008/09 New Zealand adult nutrition survey. Wellington: Ministry of Health. |

^a^Many earlier publications from NHANES are available. This reference provides data standardised for assay differences

**Table S5: Large ongoing randomised controlled trials of vitamin D supplements with relevant prior 25-hydroxyvitamin D surveys**

| **Study** | **Reference for protocol information (P)/relevant survey (S)** |
| --- | --- |
| **D-Health**  ACTRN12613000743763 | P: Neale RE, Armstrong BK, Baxter C, Duarte Romero B, Ebeling P, English DR, Kimlin MG, McLeod DS, O′Connell RL, van der Pols JC, Venn AJ, Webb PM, Whiteman DC, Wockner L. The D-Health Trial: A randomized trial of vitamin D for prevention of mortality and cancer. Contemp Clin Trials 2016;48:83-90. |
|  | S: Tran B, Armstrong BK, Carlin JB, Ebeling PR, English DR, Kimlin MG, Rahman B, van der Pols JC, Venn A, Gebski V, Whiteman DC, Webb PM, Neale RE. Recruitment and results of a pilot trial of vitamin D supplementation in the general population of Australia. J Clin Endocrinol Metab 2012;97:4473-80  S:Waterhouse M, Tran B, Ebeling PR, English DR, Lucas RM, Venn AJ, Webb PM, Whiteman DC, Neale RE. Effect of vitamin D supplementation on selected inflammatory biomarkers in older adults: a secondary analysis of data from a randomised, placebo-controlled trial. Br J Nutr 2015;114:693-9. |
|  | S:Australian Health Survey 2011 – 2012  <http://www.abs.gov.au/AUSSTATS/abs@.nsf/DetailsPage/4364.0.55.0062011-12?OpenDocument> |
| **DO-HEALTH**  NCT01745263 | P:Nil |
| **Finnish Vitamin D Trial**  NCT01463813 | P:<http://www2.uef.fi/documents/1129027/0/FIND+research+plan/3c1b9701-52f0-4ef8-989e-c56d5bc52b1c> |
|  | S: Hurskainen AR, Virtanen JK, Tuomainen TP, Nurmi T, Voutilianen S. Association of serum 25-hydroxyvitamin D with type 2 diabetes and markers of insulin resistance in a general older population in Finland. Diabetes/Metab Research Reviews 2012;28:418-23 |
|  | S;Carlberg C, Seuter S, de Mello VD, Schwab U, Voutilainen S, Pulkki K, Nurmi T, Virtanen J, Toumainen TP, Uusitupa M. Primary vitamin D target genes allow a categorization of possible benefits of vitamin D_3_ supplementation. PLoS ONE 8(&):e71042 |
|  | S:Salminen M, Saaristo P, Salonoja M, Vaapio S, Vahlberg T, Lamberg-Allardt C, Aarnio P, Kivela S-L. Arch Gerontol Geriatric 2015;61:419-24 |
| **International Polycap Study (TIPS-3)**  NCT01646437 | P:Nil |
| **VITAL**  NCT01169259 | P:Manson JA, Bassuk SS, Lee IM, Cook NR, Albert MA, Gordon D, Zaharris E, MacFadyen JG, Danielson E, Lin J, Zhang SM, Buring JE. The VITamin D and OmegA-3 TriaL (VITAL): Rationale and design of a large randomized controlled trial of vitamin D and marine omega-3 fatty acid supplements for the primary prevention of cancer and cardiovascular disease. Contemp Clin Trials 2012;33:159-71.  S:Looker AC, Dawson-Hughes B, Calvo MS, Gunter EW, Sahyoun NR. Serum 25-hydroxyvitamin D status of adolescents and adults in two seasonal subpopulations from NHANES III. Bone 2002;30:771-77. |
|  | S:^a^Schleicher RL, Sternberg MR, Lacher DA, Sempos CT, Looker AC, Durazo-Arvizu RA, Yetley EA, Chaudhary-Webb M, Maw KL, Pfeiffer CM, Johnson CL. The vitamin D status of the US population from 1988 to 2010 using standardized serum concentrations of 25-hydroxyvitamin D shows recent modest increases. Am J Clin Nutr 2016;104:454-61. |
| **CAPS**  NCT01052051 | P:Nil  S:^a^Schleicher RL, Sternberg MR, Lacher DA, Sempos CT, Looker AC, Durazo-Arvizu RA, Yetley EA, Chaudhary-Webb M, Maw KL, Pfeiffer CM, Johnson CL. The vitamin D status of the US population from 1988 to 2010 using standardized serum concentrations of 25-hydroxyvitamin D shows recent modest increases. Am J Clin Nutr 2016;104:454-61. |
|  | S:NHANES 2001 – 2006  <http://www.cdc.gov/nchs/products/databriefs/db59.htm> |
| **VIDAL**  ISRCTN46328341 | P:<http://www.nets.nihr.ac.uk/__data/assets/pdf_file/0003/52086/PRO-08-116-48.pdf>S: Hirani V, Primatesta P. Vitamin D concentrations among people aged 65 years and over living in private households and institutions in England: population survey. Age Ageing 2005; 34:485-91 |

^a^Many earlier publications from NHANES are available. This reference provides data standardised for assay differences

**Figure S1:** flow of studies

RCT- randomised controlled trial. One trial (Sato et al. Cerebrovasc Dis 2005;20:187-92) was excluded because multiple other trials from this group have been recently retracted due to concerns about fraudulent data.

**References**

**1.** Abou-Raya A, Abou-Raya S, Helmii M. The effect of vitamin D supplementation on inflammatory and hemostatic markers and disease activity in patients with systemic lupus erythematosus: a randomized placebo-controlled trial. *J Rheumatol.* 2013;40(3):265-272.

**2.** Aflatoonian A, Arabjahvani F, Eftekhar M, Sayadi M. Effect of vitamin D insufficiency treatment on fertility outcomes in frozen-thawed embryo transfer cycles: A randomized clinical trial. *Iran J Reprod Med.* 2014;12(9):595-600.

**3.** Aloia JF, Li-Ng M. Re: epidemic influenza and vitamin D. *Epidemiol Infect.* 2007;135(7):1095-1096; author reply 1097-1098.

**4.** Amestejani M, Salehi BS, Vasigh M, et al. Vitamin D supplementation in the treatment of atopic dermatitis: a clinical trial study. *J Drugs Dermatol.* 2012;11(3):327-330.

**5.** Aminmansour B, Nikbakht H, Ghorbani A, et al. Comparison of the administration of progesterone versus progesterone and vitamin D in improvement of outcomes in patients with traumatic brain injury: A randomized clinical trial with placebo group. *Adv Biomed Res.* 2012;1:58.

**6.** Amrein K, Schnedl C, Holl A, et al. Effect of high-dose vitamin D3 on hospital length of stay in critically ill patients with vitamin D deficiency: the VITdAL-ICU randomized clinical trial. *JAMA.* 2014;312(15):1520-1530.

**7.** Andreoli L, Dall'Ara F, Piantoni S, et al. A 24-month prospective study on the efficacy and safety of two different monthly regimens of vitamin D supplementation in pre-menopausal women with systemic lupus erythematosus. *Lupus.* 2015;24(4-5):499-506.

**8.** Arvold DS, Odean MJ, Dornfeld MP, et al. Correlation of symptoms with vitamin D deficiency and symptom response to cholecalciferol treatment: a randomized controlled trial. *Endocr Pract.* 2009;15(3):203-212.

**9.** Asadi M, Matin N, Frootan M, Mohamadpour J, Qorbani M, Tanha FD. Vitamin D improves endometrial thickness in PCOS women who need intrauterine insemination: a randomized double-blind placebo-controlled trial. *Arch Gynecol Obstet.* 2014;289(4):865-870.

**10.** Asemi Z, Karamali M, Esmaillzadeh A. Favorable effects of vitamin D supplementation on pregnancy outcomes in gestational diabetes: a double blind randomized controlled clinical trial. *Horm Metab Res.* 2015;47(8):565-570.

**11.** Baron JA, Barry EL, Mott LA, et al. A Trial of Calcium and Vitamin D for the Prevention of Colorectal Adenomas. *N Engl J Med.* 2015;373(16):1519-1530.

**12.** Bergman P, Norlin AC, Hansen S, et al. Vitamin D3 supplementation in patients with frequent respiratory tract infections: a randomised and double-blind intervention study. *BMJ Open.* 2012;2(6).

**13.** Bhan I, Dobens D, Tamez H, et al. Nutritional vitamin D supplementation in dialysis: a randomized trial. *Clin J Am Soc Nephrol.* 2015;10(4):611-619.

**14.** Bischoff HA, Stahelin HB, Dick W, et al. Effects of vitamin D and calcium supplementation on falls: a randomized controlled trial. *J Bone Miner Res.* 2003;18(2):343-351.

**15.** Bischoff-Ferrari HA, Dawson-Hughes B, Platz A, et al. Effect of high-dosage cholecalciferol and extended physiotherapy on complications after hip fracture: a randomized controlled trial. *Arch Intern Med.* 2010;170(9):813-820.

**16.** Bjorkman M, Sorva A, Tilvis R. Vitamin D supplementation has no major effect on pain or pain behavior in bedridden geriatric patients with advanced dementia. *Aging Clin Exp Res.* 2008;20(4):316-321.

**17.** Broe KE, Chen TC, Weinberg J, Bischoff-Ferrari HA, Holick MF, Kiel DP. A higher dose of vitamin d reduces the risk of falls in nursing home residents: a randomized, multiple-dose study. *J Am Geriatr Soc.* 2007;55(2):234-239.

**18.** Brohult J, Jonson B. Effects of large doses of calciferol on patients with rheumatoid arthritis. A double-blind clinical trial. *Scand J Rheumatol.* 1973;2(4):173-176.

**19.** Brooke OG, Brown IR, Bone CD, et al. Vitamin D supplements in pregnant Asian women: effects on calcium status and fetal growth. *Br Med J.* 1980;280(6216):751-754.

**20.** Burkiewicz CJ, Guadagnin FA, Skare TL, do Nascimento MM, Servin SC, de Souza GD. Vitamin D and skin repair: a prospective, double-blind and placebo controlled study in the healing of leg ulcers. *Rev Col Bras Cir.* 2012;39(5):401-407.

**21.** Burleigh E, McColl J, Potter J. Does vitamin D stop inpatients falling? A randomised controlled trial. *Age Ageing.* 2007;36(5):507-513.

**22.** Burton JM, Kimball S, Vieth R, et al. A phase I/II dose-escalation trial of vitamin D3 and calcium in multiple sclerosis. *Neurology.* 2010;74(23):1852-1859.

**23.** Cangussu LM, Nahas-Neto J, Orsatti CL, et al. Effect of isolated vitamin D supplementation on the rate of falls and postural balance in postmenopausal women fallers: a randomized, double-blind, placebo-controlled trial. *Menopause.* 2015.

**24.** Castro M, King TS, Kunselman SJ, et al. Effect of vitamin D3 on asthma treatment failures in adults with symptomatic asthma and lower vitamin D levels: the VIDA randomized clinical trial. *JAMA.* 2014;311(20):2083-2091.

**25.** Catalano A, Morabito N, Atteritano M, Basile G, Cucinotta D, Lasco A. Vitamin D reduces musculoskeletal pain after infusion of zoledronic acid for postmenopausal osteoporosis. *Calcif Tissue Int.* 2012;90(4):279-285.

**26.** Chapuy MC, Arlot ME, Delmas PD, Meunier PJ. Effect of calcium and cholecalciferol treatment for three years on hip fractures in elderly women. *BMJ.* 1994;308(6936):1081-1082.

**27.** Chapuy MC, Pamphile R, Paris E, et al. Combined calcium and vitamin D3 supplementation in elderly women: confirmation of reversal of secondary hyperparathyroidism and hip fracture risk: the Decalyos II study. *Osteoporos Int.* 2002;13(3):257-264.

**28.** Christiansen C, Rodbro P, Sjo O. "Anticonvulsant action" of vitamin D in epileptic patients? A controlled pilot study. *Br Med J.* 1974;2(5913):258-259.

**29.** Daley P, Jagannathan V, John KR, et al. Adjunctive vitamin D for treatment of active tuberculosis in India: a randomised, double-blind, placebo-controlled trial. *Lancet Infect Dis.* 2015;15(5):528-534.

**30.** Davidson MB, Duran P, Lee ML, Friedman TC. High-dose vitamin D supplementation in people with prediabetes and hypovitaminosis D. *Diabetes Care.* 2013;36(2):260-266.

**31.** Dawson-Hughes B, Harris SS, Krall EA, Dallal GE. Effect of calcium and vitamin D supplementation on bone density in men and women 65 years of age or older. *N Engl J Med.* 1997;337(10):670-676.

**32.** de Gruijl FR, Pavel S. The effects of a mid-winter 8-week course of sub-sunburn sunbed exposures on tanning, vitamin D status and colds. *Photochem Photobiol Sci.* 2012;11(12):1848-1854.

**33.** Dehghan A, Rahimpour S, Soleymani-Salehabadi H, Owlia MB. Role of vitamin D in flare ups of rheumatoid arthritis. *Z Rheumatol.* 2014;73(5):461-464.

**34.** Deng XL, Li YM, Yang XY, Huang JR, Guo SL, Song LM. [Efficacy and safety of vitamin D in the treatment of idiopathic oligoasthenozoospermia]. *Zhonghua Nan Ke Xue.* 2014;20(12):1082-1085.

**35.** Derakhshandi H, Etemadifar M, Feizi A, et al. Preventive effect of vitamin D3 supplementation on conversion of optic neuritis to clinically definite multiple sclerosis: a double blind, randomized, placebo-controlled pilot clinical trial. *Acta Neurol Belg.* 2013;113(3):257-263.

**36.** Dutta D, Mondal SA, Choudhuri S, et al. Vitamin-D supplementation in prediabetes reduced progression to type 2 diabetes and was associated with decreased insulin resistance and systemic inflammation: an open label randomized prospective study from Eastern India. *Diabetes Res Clin Pract.* 2014;103(3):e18-23.

**37.** Etemadifar M, Janghorbani M. Efficacy of high-dose vitamin D3 supplementation in vitamin D deficient pregnant women with multiple sclerosis: Preliminary findings of a randomized-controlled trial. *Iran J Neurol.* 2015;14(2):67-73.

**38.** Firouzabadi R, Aflatoonian A, Modarresi S, Sekhavat L, MohammadTaheri S. Therapeutic effects of calcium & vitamin D supplementation in women with PCOS. *Complement Ther Clin Pract.* 2012;18(2):85-88.

**39.** Flicker L, MacInnis RJ, Stein MS, et al. Should older people in residential care receive vitamin D to prevent falls? Results of a randomized trial. *J Am Geriatr Soc.* 2005;53(11):1881-1888.

**40.** Frandsen TB, Pareek M, Hansen JP, Nielsen CT. Vitamin D supplementation for treatment of seasonal affective symptoms in healthcare professionals: a double-blind randomised placebo-controlled trial. *BMC Res Notes.* 2014;7:528.

**41.** Gendelman O, Itzhaki D, Makarov S, Bennun M, Amital H. A randomized double-blind placebo-controlled study adding high dose vitamin D to analgesic regimens in patients with musculoskeletal pain. *Lupus.* 2015;24(4-5):483-489.

**42.** Glendenning P, Zhu K, Inderjeeth C, Howat P, Lewis JR, Prince RL. Effects of three-monthly oral 150,000 IU cholecalciferol supplementation on falls, mobility, and muscle strength in older postmenopausal women: a randomized controlled trial. *J Bone Miner Res.* 2012;27(1):170-176.

**43.** Golan D, Halhal B, Glass-Marmor L, et al. Vitamin D supplementation for patients with multiple sclerosis treated with interferon-beta: a randomized controlled trial assessing the effect on flu-like symptoms and immunomodulatory properties. *BMC Neurol.* 2013;13:60.

**44.** Goldring ST, Griffiths CJ, Martineau AR, et al. Prenatal vitamin d supplementation and child respiratory health: a randomised controlled trial. *PLoS One.* 2013;8(6):e66627.

**45.** Goodall EC, Granados AC, Luinstra K, et al. Vitamin D3 and gargling for the prevention of upper respiratory tract infections: a randomized controlled trial. *BMC Infect Dis.* 2014;14:273.

**46.** Grant AM, Avenell A, Campbell MK, et al. Oral vitamin D3 and calcium for secondary prevention of low-trauma fractures in elderly people (Randomised Evaluation of Calcium Or vitamin D, RECORD): a randomised placebo-controlled trial. *Lancet.* 2005;365(9471):1621-1628.

**47.** Grant CC, Kaur S, Waymouth E, et al. Reduced primary care respiratory infection visits following pregnancy and infancy vitamin D supplementation: a randomised controlled trial. *Acta Paediatr.* 2015;104(4):396-404.

**48.** Grossmann RE, Zughaier SM, Kumari M, et al. Pilot study of vitamin D supplementation in adults with cystic fibrosis pulmonary exacerbation: A randomized, controlled trial. *Dermatoendocrinol.* 2012;4(2):191-197.

**49.** Hansen KE, Bartels CM, Gangnon RE, Jones AN, Gogineni J. An evaluation of high-dose vitamin D for rheumatoid arthritis. *J Clin Rheumatol.* 2014;20(2):112-114.

**50.** Harwood RH, Sahota O, Gaynor K, Masud T, Hosking DJ, Nottingham Neck of Femur S. A randomised, controlled comparison of different calcium and vitamin D supplementation regimens in elderly women after hip fracture: The Nottingham Neck of Femur (NONOF) Study. *Age Ageing.* 2004;33(1):45-51.

**51.** Hata TR, Audish D, Kotol P, et al. A randomized controlled double-blind investigation of the effects of vitamin D dietary supplementation in subjects with atopic dermatitis. *J Eur Acad Dermatol Venereol.* 2014;28(6):781-789.

**52.** Helou J, Moutran R, Maatouk I, Haddad F. Raynaud's phenomenon and vitamin D. *Rheumatol Int.* 2013;33(3):751-755.

**53.** Hiremath VP, Rao CB, Naik V, Prasad KV. Anti-inflammatory effect of vitamin D on gingivitis: a dose-response randomised control trial. *Oral Health Prev Dent.* 2013;11(1):61-69.

**54.** Hollis BW, Johnson D, Hulsey TC, Ebeling M, Wagner CL. Vitamin D supplementation during pregnancy: double-blind, randomized clinical trial of safety and effectiveness. *J Bone Miner Res.* 2011;26(10):2341-2357.

**55.** Hossain N, Kanani FH, Ramzan S, et al. Obstetric and neonatal outcomes of maternal vitamin D supplementation: results of an open-label, randomized controlled trial of antenatal vitamin D supplementation in Pakistani women. *J Clin Endocrinol Metab.* 2014;99(7):2448-2455.

**56.** Houston DK, Tooze JA, Demons JL, et al. Delivery of a Vitamin D Intervention in Homebound Older Adults Using a Meals-on-Wheels Program: A Pilot Study. *J Am Geriatr Soc.* 2015;63(9):1861-1867.

**57.** Inkovaara J, Gothoni G, Halttula R, Heikinheimo R, Tokola O. Calcium, vitamin D and anabolic steroid in treatment of aged bones: double-blind placebo-controlled long-term clinical trial. *Age Ageing.* 1983;12(2):124-130.

**58.** Jackson RD, LaCroix AZ, Gass M, et al. Calcium plus vitamin D supplementation and the risk of fractures. *N Engl J Med.* 2006;354(7):669-683.

**59.** Jorde R, Sneve M, Figenschau Y, Svartberg J, Waterloo K. Effects of vitamin D supplementation on symptoms of depression in overweight and obese subjects: randomized double blind trial. *J Intern Med.* 2008;264(6):599-609.

**60.** Jorgensen SP, Agnholt J, Glerup H, et al. Clinical trial: vitamin D3 treatment in Crohn's disease - a randomized double-blind placebo-controlled study. *Aliment Pharmacol Ther.* 2010;32(3):377-383.

**61.** Kampman MT, Steffensen LH, Mellgren SI, Jorgensen L. Effect of vitamin D3 supplementation on relapses, disease progression, and measures of function in persons with multiple sclerosis: exploratory outcomes from a double-blind randomised controlled trial. *Mult Scler.* 2012;18(8):1144-1151.

**62.** Karamali M, Asemi Z, Ahmadi-Dastjerdi M, Esmaillzadeh A. Calcium plus vitamin D supplementation affects pregnancy outcomes in gestational diabetes: randomized, double-blind, placebo-controlled trial. *Public Health Nutr.* 2015:1-8.

**63.** Khajehei M, Abdali K, Tabatabaee HR. A comparison between the efficacy of dydrogesterone and calcium plus vitamin D in improving women's general health. *Afr J Psychiatry (Johannesbg).* 2010;13(3):218-224.

**64.** Khoraminya N, Tehrani-Doost M, Jazayeri S, Hosseini A, Djazayery A. Therapeutic effects of vitamin D as adjunctive therapy to fluoxetine in patients with major depressive disorder. *Aust N Z J Psychiatry.* 2013;47(3):271-275.

**65.** Kjaergaard M, Waterloo K, Wang CE, et al. Effect of vitamin D supplement on depression scores in people with low levels of serum 25-hydroxyvitamin D: nested case-control study and randomised clinical trial. *Br J Psychiatry.* 2012;201(5):360-368.

**66.** Knutsen KV, Madar AA, Brekke M, et al. Effect of vitamin D on musculoskeletal pain and headache: A randomized, double-blind, placebo-controlled trial among adult ethnic minorities in Norway. *Pain.* 2014;155(12):2591-2598.

**67.** Komulainen MH, Kroger H, Tuppurainen MT, et al. HRT and Vit D in prevention of non-vertebral fractures in postmenopausal women; a 5 year randomized trial. *Maturitas.* 1998;31(1):45-54.

**68.** Kota SK, Jammula S, Kota SK, Tripathy PR, Panda S, Modi KD. Effect of vitamin D supplementation in type 2 diabetes patients with pulmonary tuberculosis. *Diabetes Metab Syndr.* 2011;5(2):85-89.

**69.** Kuchay MS, Laway BA, Bashir MI, Wani AI, Misgar RA, Shah ZA. Effect of Vitamin D supplementation on glycemic parameters and progression of prediabetes to diabetes: A 1-year, open-label randomized study. *Indian J Endocrinol Metab.* 2015;19(3):387-392.

**70.** Laaksi I, Ruohola JP, Mattila V, Auvinen A, Ylikomi T, Pihlajamaki H. Vitamin D supplementation for the prevention of acute respiratory tract infection: a randomized, double-blinded trial among young Finnish men. *J Infect Dis.* 2010;202(5):809-814.

**71.** Lappe JM, Travers-Gustafson D, Davies KM, Recker RR, Heaney RP. Vitamin D and calcium supplementation reduces cancer risk: results of a randomized trial. *Am J Clin Nutr.* 2007;85(6):1586-1591.

**72.** Lappe J, Cullen D, Haynatzki G, Recker R, Ahlf R, Thompson K. Calcium and vitamin d supplementation decreases incidence of stress fractures in female navy recruits. *J Bone Miner Res.* 2008;23(5):741-749.

**73.** Larsen ER, Mosekilde L, Foldspang A. Vitamin D and calcium supplementation prevents osteoporotic fractures in elderly community dwelling residents: a pragmatic population-based 3-year intervention study. *J Bone Miner Res.* 2004;19(3):370-378.

**74.** Lasco A, Catalano A, Benvenga S. Improvement of primary dysmenorrhea caused by a single oral dose of vitamin D: results of a randomized, double-blind, placebo-controlled study. *Arch Intern Med.* 2012;172(4):366-367.

**75.** Latham NK, Anderson CS, Lee A, et al. A randomized, controlled trial of quadriceps resistance exercise and vitamin D in frail older people: the Frailty Interventions Trial in Elderly Subjects (FITNESS). *J Am Geriatr Soc.* 2003;51(3):291-299.

**76.** Law M, Withers H, Morris J, Anderson F. Vitamin D supplementation and the prevention of fractures and falls: results of a randomised trial in elderly people in residential accommodation. *Age Ageing.* 2006;35(5):482-486.

**77.** Lehouck A, Mathieu C, Carremans C, et al. High doses of vitamin D to reduce exacerbations in chronic obstructive pulmonary disease: a randomized trial. *Ann Intern Med.* 2012;156(2):105-114.

**78.** Li-Ng M, Aloia JF, Pollack S, et al. A randomized controlled trial of vitamin D3 supplementation for the prevention of symptomatic upper respiratory tract infections. *Epidemiol Infect.* 2009;137(10):1396-1404.

**79.** Lips P, Graafmans WC, Ooms ME, Bezemer PD, Bouter LM. Vitamin D supplementation and fracture incidence in elderly persons. A randomized, placebo-controlled clinical trial. *Ann Intern Med.* 1996;124(4):400-406.

**80.** Lopez-Torres Hidalgo J, Grupo A. [Effect of calcium and vitamin D in the reduction of falls in the elderly: a randomized trial versus placebo]. *Med Clin (Barc).* 2014;142(3):95-102.

**81.** Lyons RA, Johansen A, Brophy S, et al. Preventing fractures among older people living in institutional care: a pragmatic randomised double blind placebo controlled trial of vitamin D supplementation. *Osteoporos Int.* 2007;18(6):811-818.

**82.** Martineau AR, Timms PM, Bothamley GH, et al. High-dose vitamin D(3) during intensive-phase antimicrobial treatment of pulmonary tuberculosis: a double-blind randomised controlled trial. *Lancet.* 2011;377(9761):242-250.

**83.** Martineau AR, MacLaughlin BD, Hooper RL, et al. Double-blind randomised placebo-controlled trial of bolus-dose vitamin D3 supplementation in adults with asthma (ViDiAs). *Thorax.* 2015;70(5):451-457.

**84.** Martineau AR, Hanifa Y, Witt KD, et al. Double-blind randomised controlled trial of vitamin D3 supplementation for the prevention of acute respiratory infection in older adults and their carers (ViDiFlu). *Thorax.* 2015;70(10):953-960.

**85.** Martineau AR, James WY, Hooper RL, et al. Vitamin D3 supplementation in patients with chronic obstructive pulmonary disease (ViDiCO): a multicentre, double-blind, randomised controlled trial. *Lancet Respir Med.* 2015;3(2):120-130.

**86.** Marya RK, Rathee S, Manrow M. Effect of calcium and vitamin D supplementation on toxaemia of pregnancy. *Gynecol Obstet Invest.* 1987;24(1):38-42.

**87.** McAlindon T, LaValley M, Schneider E, et al. Effect of vitamin D supplementation on progression of knee pain and cartilage volume loss in patients with symptomatic osteoarthritis: a randomized controlled trial. *JAMA.* 2013;309(2):155-162.

**88.** Meyer HE, Smedshaug GB, Kvaavik E, Falch JA, Tverdal A, Pedersen JI. Can vitamin D supplementation reduce the risk of fracture in the elderly? A randomized controlled trial. *J Bone Miner Res.* 2002;17(4):709-715.

**89.** Miskulin DC, Majchrzak K, Tighiouart H, et al. Ergocalciferol Supplementation in Hemodialysis Patients With Vitamin D Deficiency: A Randomized Clinical Trial. *J Am Soc Nephrol.* 2015.

**90.** Mosayebi G, Ghazavi A, Ghasami K, Jand Y, Kokhaei P. Therapeutic effect of vitamin D3 in multiple sclerosis patients. *Immunol Invest.* 2011;40(6):627-639.

**91.** Mottaghi T, Askari G, Khorvash F, Maracy MR. Effect of Vitamin D supplementation on symptoms and C-reactive protein in migraine patients. *J Res Med Sci.* 2015;20(5):477-482.

**92.** Mozaffari-Khosravi H, Nabizade L, Yassini-Ardakani SM, Hadinedoushan H, Barzegar K. The effect of 2 different single injections of high dose of vitamin D on improving the depression in depressed patients with vitamin D deficiency: a randomized clinical trial. *J Clin Psychopharmacol.* 2013;33(3):378-385.

**93.** Murdoch DR, Slow S, Chambers ST, et al. Effect of vitamin D3 supplementation on upper respiratory tract infections in healthy adults: the VIDARIS randomized controlled trial. *JAMA.* 2012;308(13):1333-1339.

**94.** Nair P, Venkatesh B, Lee P, et al. A Randomized Study of a Single Dose of Intramuscular Cholecalciferol in Critically Ill Adults. *Crit Care Med.* 2015;43(11):2313-2320.

**95.** Nursyam EW, Amin Z, Rumende CM. The effect of vitamin D as supplementary treatment in patients with moderately advanced pulmonary tuberculous lesion. *Acta Med Indones.* 2006;38(1):3-5.

**96.** Pfeifer M, Begerow B, Minne HW, Abrams C, Nachtigall D, Hansen C. Effects of a short-term vitamin D and calcium supplementation on body sway and secondary hyperparathyroidism in elderly women. *J Bone Miner Res.* 2000;15(6):1113-1118.

**97.** Pfeifer M, Begerow B, Minne HW, Suppan K, Fahrleitner-Pammer A, Dobnig H. Effects of a long-term vitamin D and calcium supplementation on falls and parameters of muscle function in community-dwelling older individuals. *Osteoporos Int.* 2009;20(2):315-322.

**98.** Porthouse J, Cockayne S, King C, et al. Randomised controlled trial of calcium and supplementation with cholecalciferol (vitamin D3) for prevention of fractures in primary care. *BMJ.* 2005;330(7498):1003.

**99.** Prince RL, Austin N, Devine A, Dick IM, Bruce D, Zhu K. Effects of ergocalciferol added to calcium on the risk of falls in elderly high-risk women. *Arch Intern Med.* 2008;168(1):103-108.

**100.** Punthakee Z, Bosch J, Dagenais G, et al. Design, history and results of the Thiazolidinedione Intervention with vitamin D Evaluation (TIDE) randomised controlled trial. *Diabetologia.* 2012;55(1):36-45.

**101.** Raftery T, Martineau AR, Greiller CL, et al. Effects of vitamin D supplementation on intestinal permeability, cathelicidin and disease markers in Crohn's disease: Results from a randomised double-blind placebo-controlled study. *United European Gastroenterol J.* 2015;3(3):294-302.

**102.** Ralph AP, Waramori G, Pontororing GJ, et al. L-arginine and vitamin D adjunctive therapies in pulmonary tuberculosis: a randomised, double-blind, placebo-controlled trial. *PLoS One.* 2013;8(8):e70032.

**103.** Rashidi B, Haghollahi F, Shariat M, Zayerii F. The effects of calcium-vitamin D and metformin on polycystic ovary syndrome: a pilot study. *Taiwan J Obstet Gynecol.* 2009;48(2):142-147.

**104.** Rastelli AL, Taylor ME, Gao F, et al. Vitamin D and aromatase inhibitor-induced musculoskeletal symptoms (AIMSS): a phase II, double-blind, placebo-controlled, randomized trial. *Breast Cancer Res Treat.* 2011;129(1):107-116.

**105.** Rizzoli R, Dawson-Hughes B, Kaufman JM, et al. Correction of vitamin D insufficiency with combined strontium ranelate and vitamin D3 in osteoporotic patients. *Eur J Endocrinol.* 2014;170(3):441-450.

**106.** Rorie A, Goldner WS, Lyden E, Poole JA. Beneficial role for supplemental vitamin D3 treatment in chronic urticaria: a randomized study. *Ann Allergy Asthma Immunol.* 2014;112(4):376-382.

**107.** Roth DE, Al Mahmud A, Raqib R, et al. Randomized placebo-controlled trial of high-dose prenatal third-trimester vitamin D3 supplementation in Bangladesh: the AViDD trial. *Nutr J.* 2013;12:47.

**108.** Sablok A, Batra A, Thariani K, et al. Supplementation of vitamin D in pregnancy and its correlation with feto-maternal outcome. *Clin Endocrinol (Oxf).* 2015;83(4):536-541.

**109.** Sakalli H, Arslan D, Yucel AE. The effect of oral and parenteral vitamin D supplementation in the elderly: a prospective, double-blinded, randomized, placebo-controlled study. *Rheumatol Int.* 2012;32(8):2279-2283.

**110.** Salahuddin N, Ali F, Hasan Z, Rao N, Aqeel M, Mahmood F. Vitamin D accelerates clinical recovery from tuberculosis: results of the SUCCINCT Study [Supplementary Cholecalciferol in recovery from tuberculosis]. A randomized, placebo-controlled, clinical trial of vitamin D supplementation in patients with pulmonary tuberculosis'. *BMC Infect Dis.* 2013;13:22.

**111.** Salesi M, Farajzadegan Z. Efficacy of vitamin D in patients with active rheumatoid arthritis receiving methotrexate therapy. *Rheumatol Int.* 2012;32(7):2129-2133.

**112.** Salovaara K, Tuppurainen M, Karkkainen M, et al. Effect of vitamin D(3) and calcium on fracture risk in 65- to 71-year-old women: a population-based 3-year randomized, controlled trial--the OSTPRE-FPS. *J Bone Miner Res.* 2010;25(7):1487-1495.

**113.** Sanders KM, Stuart AL, Williamson EJ, et al. Annual high-dose oral vitamin D and falls and fractures in older women: a randomized controlled trial. *JAMA.* 2010;303(18):1815-1822.

**114.** Sandoughi M, Zakeri Z, Mirhosainee Z, Mohammadi M, Shahbakhsh S. The effect of vitamin D on nonspecific low back pain. *Int J Rheum Dis.* 2015;18(8):854-858.

**115.** Sanghi D, Mishra A, Sharma AC, et al. Does vitamin D improve osteoarthritis of the knee: a randomized controlled pilot trial. *Clin Orthop Relat Res.* 2013;471(11):3556-3562.

**116.** Schleithoff SS, Zittermann A, Tenderich G, Berthold HK, Stehle P, Koerfer R. Vitamin D supplementation improves cytokine profiles in patients with congestive heart failure: a double-blind, randomized, placebo-controlled trial. *Am J Clin Nutr.* 2006;83(4):754-759.

**117.** Schreuder F, Bernsen RM, van der Wouden JC. Vitamin D supplementation for nonspecific musculoskeletal pain in non-Western immigrants: a randomized controlled trial. *Ann Fam Med.* 2012;10(6):547-555.

**118.** Sepehrmanesh Z, Kolahdooz F, Abedi F, et al. Vitamin D Supplementation Affects the Beck Depression Inventory, Insulin Resistance, and Biomarkers of Oxidative Stress in Patients with Major Depressive Disorder: A Randomized, Controlled Clinical Trial. *J Nutr.* 2015.

**119.** Shirazian S, Schanler M, Shastry S, et al. The effect of ergocalciferol on uremic pruritus severity: a randomized controlled trial. *J Ren Nutr.* 2013;23(4):308-314.

**120.** Smith H, Anderson F, Raphael H, Maslin P, Crozier S, Cooper C. Effect of annual intramuscular vitamin D on fracture risk in elderly men and women--a population-based, randomized, double-blind, placebo-controlled trial. *Rheumatology (Oxford).* 2007;46(12):1852-1857.

**121.** Souwer IH, Lagro-Janssen AL. Vitamin D3 is not effective in the treatment of chronic chilblains. *Int J Clin Pract.* 2009;63(2):282-286.

**122.** Stein MS, Liu Y, Gray OM, et al. A randomized trial of high-dose vitamin D2 in relapsing-remitting multiple sclerosis. *Neurology.* 2011;77(17):1611-1618.

**123.** Stein MS, Scherer SC, Ladd KS, Harrison LC. A randomized controlled trial of high-dose vitamin D2 followed by intranasal insulin in Alzheimer's disease. *J Alzheimers Dis.* 2011;26(3):477-484.

**124.** Suzuki M, Yoshioka M, Hashimoto M, et al. Randomized, double-blind, placebo-controlled trial of vitamin D supplementation in Parkinson disease. *Am J Clin Nutr.* 2013;97(5):1004-1013.

**125.** Tran B, Armstrong BK, Ebeling PR, et al. Effect of vitamin D supplementation on antibiotic use: a randomized controlled trial. *Am J Clin Nutr.* 2014;99(1):156-161.

**126.** Trivedi DP, Doll R, Khaw KT. Effect of four monthly oral vitamin D3 (cholecalciferol) supplementation on fractures and mortality in men and women living in the community: randomised double blind controlled trial. *BMJ.* 2003;326(7387):469.

**127.** Tukvadze N, Sanikidze E, Kipiani M, et al. High-dose vitamin D3 in adults with pulmonary tuberculosis: a double-blind randomized controlled trial. *Am J Clin Nutr.* 2015;102(5):1059-1069.

**128.** Turner AN, Carr Reese P, Fields KS, et al. A blinded, randomized controlled trial of high-dose vitamin D supplementation to reduce recurrence of bacterial vaginosis. *Am J Obstet Gynecol.* 2014;211(5):479 e471-479 e413.

**129.** Uusi-Rasi K, Patil R, Karinkanta S, et al. Exercise and vitamin D in fall prevention among older women: a randomized clinical trial. *JAMA Intern Med.* 2015;175(5):703-711.

**130.** Vieth R, Kimball S, Hu A, Walfish PG. Randomized comparison of the effects of the vitamin D3 adequate intake versus 100 mcg (4000 IU) per day on biochemical responses and the wellbeing of patients. *Nutr J.* 2004;3:8.

**131.** Wagner CL, McNeil R, Hamilton SA, et al. A randomized trial of vitamin D supplementation in 2 community health center networks in South Carolina. *Am J Obstet Gynecol.* 2013;208(2):137 e131-113.

**132.** Warner AE, Arnspiger SA. Diffuse musculoskeletal pain is not associated with low vitamin D levels or improved by treatment with vitamin D. *J Clin Rheumatol.* 2008;14(1):12-16.

**133.** Wasse H, Huang R, Long Q, et al. Very high-dose cholecalciferol and arteriovenous fistula maturation in ESRD: a randomized, double-blind, placebo-controlled pilot study. *J Vasc Access.* 2014;15(2):88-94.

**134.** Wejse C, Gomes VF, Rabna P, et al. Vitamin D as supplementary treatment for tuberculosis: a double-blind, randomized, placebo-controlled trial. *Am J Respir Crit Care Med.* 2009;179(9):843-850.

**135.** Wepner F, Scheuer R, Schuetz-Wieser B, et al. Effects of vitamin D on patients with fibromyalgia syndrome: a randomized placebo-controlled trial. *Pain.* 2014;155(2):261-268.

**136.** Witham MD, Adams F, McSwiggan S, et al. Effect of intermittent vitamin D3 on vascular function and symptoms in chronic fatigue syndrome--a randomised controlled trial. *Nutr Metab Cardiovasc Dis.* 2015;25(3):287-294.

**137.** Wood AD, Secombes KR, Thies F, et al. A parallel group double-blind RCT of vitamin D3 assessing physical function: is the biochemical response to treatment affected by overweight and obesity? *Osteoporos Int.* 2014;25(1):305-315.

**138.** Cooper C, Harvey NC, Bishop NJ, et al. Maternal gestational vitamin D supplementation and offspring bone health (MAVIDOS): a multicentre, double-blind, randomised placebo-controlled trial. *Lancet Diabetes Endocrinol.* 2016;4(5):393-402.

**139.** Scragg R, Stewart AW, Waayer D, et al. Effect of Monthly High-Dose Vitamin D Supplementation on Cardiovascular Disease in the Vitamin D Assessment Study : A Randomized Clinical Trial. *JAMA Cardiol.* 2017;2(6):608-616.
